# A modified apical resection model with high accuracy and reproducibility in neonatal mouse and rat hearts

**DOI:** 10.1038/s41536-023-00284-5

**Published:** 2023-02-18

**Authors:** Yihua Bei, Chen Chen, Xuejiao Hua, Mingming Yin, Xiangmin Meng, Zhenzhen Huang, Weitong Qi, Zhuhua Su, Chang Liu, H. Immo Lehmann, Guoping Li, Yu Huang, Junjie Xiao

**Affiliations:** 1 Cardiac Regeneration and Ageing Lab, Institute of Geriatrics (Shanghai University), Affiliated Nantong Hospital of Shanghai University (The Sixth People’s Hospital of Nantong), School of Medicine, Shanghai University, Nantong, 226011 China; 2 Institute of Cardiovascular Sciences, Shanghai Engineering Research Center of Organ Repair, School of Life Science, Shanghai University, Shanghai, 200444 China; 3 Cardiovascular Division of the Massachusetts General Hospital and Harvard Medical School, Boston, MA 02114 USA; 4 Department of Biomedical Sciences, City University of Hong Kong, Hong Kong, 999077 China

**Keywords:** Cardiac regeneration, Regenerative medicine

## Abstract

Neonatal mouse heart can regenerate after left ventricle (LV) apical resection (AR). Since current AR rodent method is accomplished by resecting LV apex until exposure of LV chamber, it is relatively difficult to operate reproducibly. We aimed to develop a modified AR method with high accuracy and reproducibility and to investigate whether cardiac regenerative capacity could be replicated in neonatal rats. For 15% AR of whole heart weight in 1-day-old (P1) neonatal mice, a modified 10 μL pipette tip cut to 0.48 mm in internal diameter was connected to a vacuum pump working at 0.06 ± 0.005 MPa and gently kept in touch with LV apex for nearly but no more than 12 s. LV apex was resected by a single incision adjacent to the pipette tip. The modified AR method in P1 mice achieved cardiac structural and functional recovery at 21 days post resection (dpr). Data from different operators showed smaller variation of resected LV apex and higher reproducibility using the modified AR method. Furthermore, we showed that 5% AR of whole heart weight in P1 neonatal rats using a modified 200 μL pipette tip cut to 0.63 mm in internal diameter led to complete regeneration of LV apex and full preservation of cardiac function at 42 dpr. In conclusion, the modified AR rodent model leads to accurate resection of LV apex with high homogeneity and reproducibility and it is practically convenient for the study of structural, functional, and molecular mechanisms of cardiac regeneration in both neonatal mice and rats.

## Introduction

The adult mammalian heart has a very limited regenerative capacity, which causes permanent myocardial loss and insufficient repair after myocardial injury^1^. Identifying molecules that promote cardiomyocyte proliferation and cardiac regeneration is essential to provide novel strategies for myocardial protection^2^. The cardiac apical resection (AR) model of neonatal mice has revealed a strong regenerative capacity of the heart within 7 days after birth^3,4^. Apical resection in 1-day-old (P1) neonatal mice induces robust cardiomyocyte proliferation and leads to cardiac regeneration with preserved cardiac function^4^. However, this regenerative capacity is lost in 7-day-old (P7) mice^4^. Based on the AR model of neonatal mice, molecules involved in postnatal cardiac regeneration have been increasingly identified which may also have the potential to promote cardiac regeneration and repair in adult hearts^5–10^. The regeneration model of neonatal mouse myocardium provides strong support for the study of cardiac regeneration in mammals.

Despite wide use of the AR model in neonatal mice, several issues remain unsolved. Although exposure of the left ventricle (LV) chamber has been used as an anatomic landmark for AR, the actual weight or size of the resected apical myocardium is difficult to control and be compared among different research groups. One reason is that either resected weight or resected area has been used to describe the resected part of apical myocardium^4,5,7,9^. Besides, since the current AR rodent method is accomplished by resecting LV apical myocardium even through several incisions^3^, it is relatively difficult to operate uniformly. Surgical technicalities and assessment procedures can vary and may result in different regeneration outcomes^4,11,12^. Therefore, a more unified and easy-to-control and easy-to-perform AR method is required.

Compared to the AR model in neonatal mice, rare studies have systematically evaluated cardiac regenerative capacity in apically resected neonatal rats. It has previously been reported that P1 rats with 16–18% apically resected area had an early cardiac repair response and long-term preserved cardiac function even under hemodynamic stress. However, the larger apically resected area in neonatal rats (16–18%) led to an incomplete regenerative process associated with myocardial hypoperfusion and collagen depositions around the repaired area at 60 days post resection (dpr)^13^. Actually, whether and how cardiac regenerative capacity could be replicated in neonatal rats remain largely unclear.

In the present study, we developed a modified AR method in neonatal mice with robust cardiac regenerative response and normal cardiac function. Compared to the conventional AR method, this modified AR method showed lower variation of resected apex and higher reproducibility of surgery. Moreover, an efficient regenerative response and structural and functional recovery of the heart were observed in P1 neonatal rats using the modified AR method. An easy-to-control and easy-to-perform AR method will be useful to promote investigation of novel mechanisms and factors for cardiac regeneration and repair.

## Results

### Modified AR method induces cardiac structural and functional recovery in neonatal mice

**Fig. 1:**
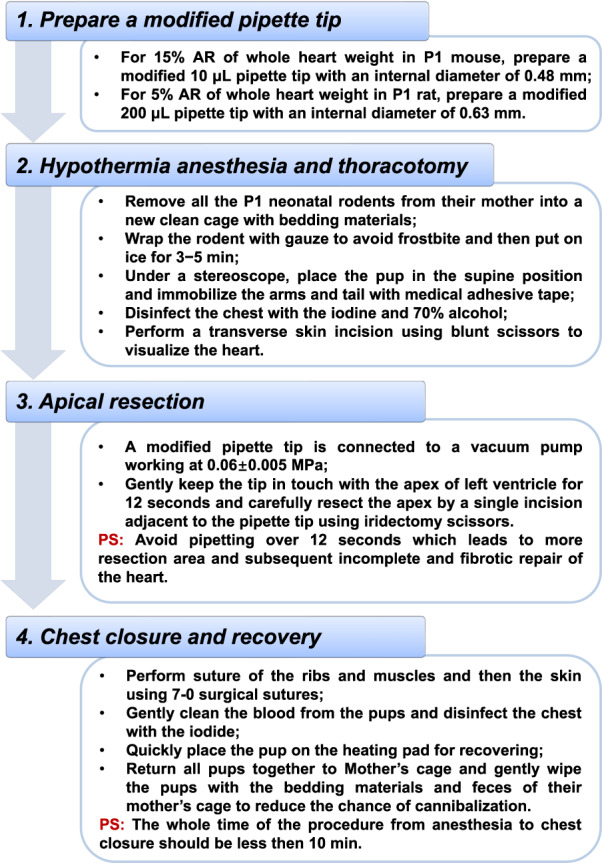
A schematic flow chart illustrating the major operation process of modified apical resection model in 1-day-old neonatal rodents. A modified pipette tip cut to a given internal diameter is prepared for the modified apical resection model. After hypothermia anesthesia and thoracotomy, the modified pipette tip, which is connected to a vacuum pump working at 0.06 ± 0.005 MPa, is gently kept in touch with the apex for 12 s. Apical resection is performed by a single incision adjacent to the pipette tip. Finally, chest closure is performed and rodents are returned together to mother’s cage.

**Fig. 2:**
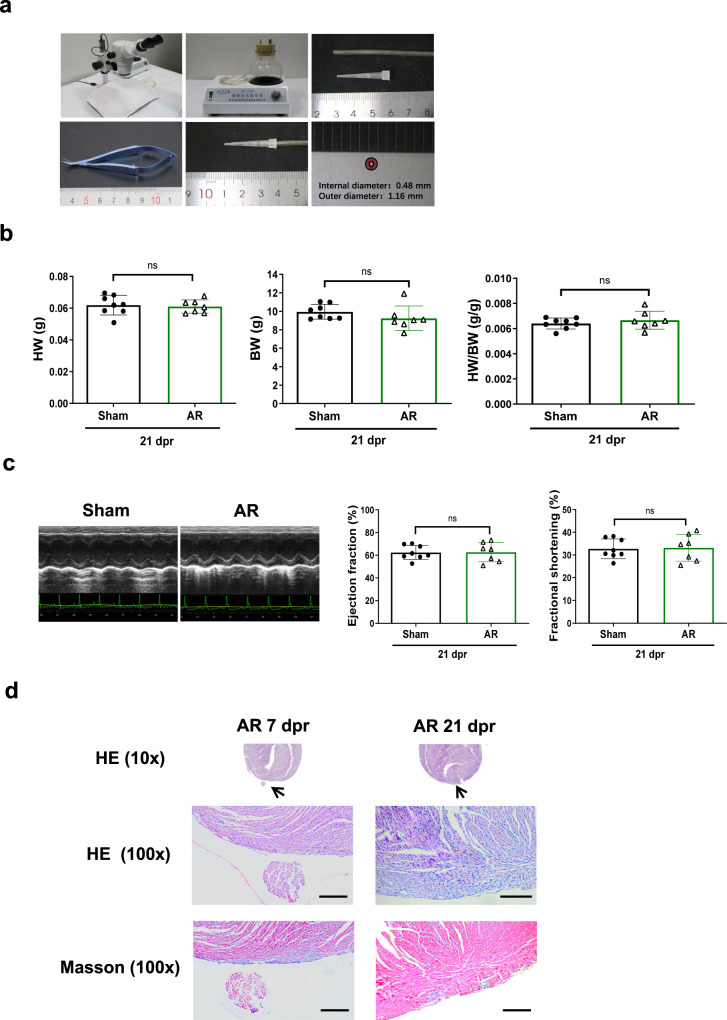
Cardiac structural and functional recovery after modified apical resection surgery in 1-day-old neonatal mice. **a** A stereoscope, a desktop vacuum pump, and a modified 10 μL pipette tip with its tip cut to 0.48 mm in internal diameter using a sharp blade, were prepared for modified apical resection (AR) method. **b** Heart weight (HW), body weight (BW), and heart weight/body weight ratio (HW/BW) at 21 days post resection (dpr) (*n* = 7-8). **c** Echocardiography for left ventricle ejection fraction and fractional shortening at 21 dpr (*n* = 7-8). **d** Representative images for hematoxylin-eosin (HE) and Masson’s trichrome staining for AR hearts at 7 dpr and 21 dpr. Scale bar = 200 μm. ns not significant. All data are expressed as means ± SD.

The modified AR method was detailed in Methods section. A schematic flow chart was presented to illustrate the major operation process of modified AR model in P1 neonatal rodents (Fig. 1). First, we tested the modified surgical AR method in P1 neonatal mice using a modified 10 μL pipette tip which was connected to a vacuum pump to accurately control the resection part of LV apex. For AR of 15% weight of P1 neonatal mouse heart, a modified 10 μL pipette tip, with its tip cut to 0.48 mm in internal diameter, was connected to a vacuum pump working at 0.06 ± 0.005 MPa (Fig. 2a). The tip was kept in touch with LV apex for 12 s and the apex was carefully resected by a single incision adjacent to the pipette tip. The weight of resected apex was proven to be 15% of whole heart weight by 3 independent experiments (Supplementary Table 1). Next, we evaluated heart weight, cardiac function, and histology of mice hearts after AR. The heart weight of AR group was already comparable to sham group at 7 dpr (Supplementary Fig. 1), and no difference was found in heart weight between AR and sham mice until 21 dpr (Fig. 2b). Echocardiography showed that AR mice had normal cardiac function that was similar to sham mice (Fig. 2c). HE staining of cardiac longitudinal sections at 7 dpr and 21 dpr revealed progressive regeneration of the apex (Fig. 2d). Masson’s trichrome staining showed that there was slight evidence of tissue fibrosis in the regenerated apex at 7 dpr, however minimal fibrosis could be observed in the apex at 21 dpr (Fig. 2d). These data indicate that this modified surgical AR method induces structural and functional recovery of the heart in P1 neonatal mice.

### Higher homogeneity and reproducibility of modified AR method compared to conventional AR method

**Fig. 3:**
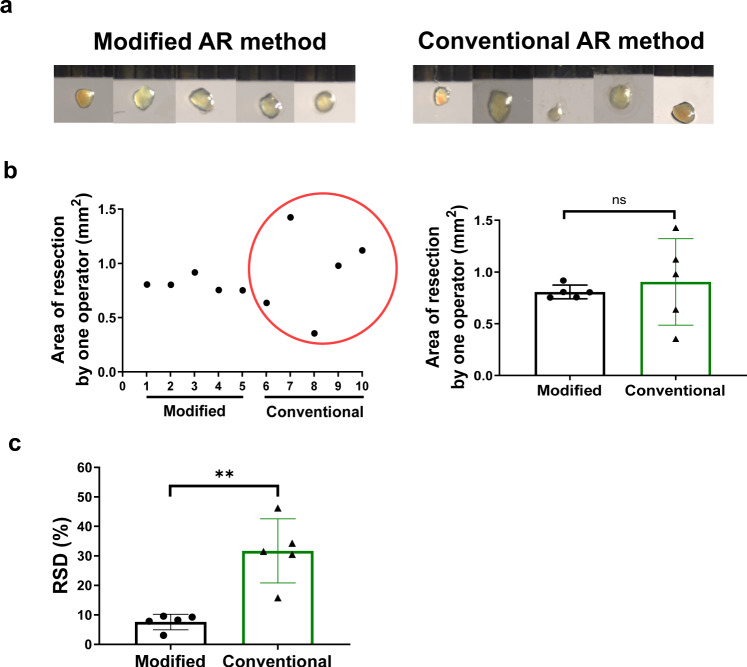
Homogeneity and reproducibility of modified and conventional apical resection methods in 1-day-old neonatal mice. **a** Representative images of resected left ventricle (LV) apex using modified apical resection (AR) method (left panel, *n* = 5) and conventional AR method (right panel, *n* = 5) by one operator. **b** Corresponding variation of resected LV apex areas (left panel) and mean value of resected apex areas using modified *versus* conventional AR methods (right panel) by the same operator. **c** Relative standard deviation (RSD) analysis for resected LV apex areas by modified *versus* conventional AR methods. **P < 0.01; ns not significant. All data are expressed as means ± SD.

To evaluate the AR operation homogeneity and reproducibility, we trained 5 operators to perform both the conventional and modified AR method. Conventional AR was accomplished by gently resecting the ventricular apex until the LV chamber was exposed^3^. The surgical AR procedure from anesthesia to chest closure should be limited within 10 min for both methods. For achieving a surgical procedure within 10 min, we observed that each operator needed to operate more mice (≈1.5 to 3 fold) when they learned and practiced conventional AR method compared to modified AR method. Then they performed formal experiments for comparison of conventional and modified AR methods. In the present study, each operator performed AR surgery in P1 neonatal mice using conventional (*n* = 5) and modified (*n* = 5) AR method, respectively. Based on the measured resected areas of LV apex, we calculated the standard deviation (SD) and relative standard deviation (RSD) in the modified or conventional AR method for each operator. Representative images of the resected apical tissue by one operator are demonstrated in Fig. 3a, showing a smaller variation of resected areas in the modified AR model compared to the conventional AR model (Fig. 3b). Compared to conventional AR method, modified AR method had lower SD and RSD of resected area of LV apex, indicating better homogeneity of resected LV apex using the modified AR method (Table 1). By further calculating the mean value of RSD from 5 operators, we observed that modified AR method achieved a significant lower RSD than conventional AR method (Fig. 3c), suggesting a higher reproducibility of the modified AR method compared to the conventional AR method.

**Table 1:** Standard deviation and relative standard deviation of the resected areas of left ventricle apex in the modified and conventional apical resection methods for each operator.

Operator	Modified AR method		Conventional AR method	
	SD (mm^2^)	RSD (%)	SD (mm^2^)	RSD (%)
1	0.0670	8.2967	0.4182	46.2493
2	0.0997	9.5579	0.3698	30.5337
3	0.1119	9.2737	0.4508	31.5350
4	0.0357	3.0897	0.1618	15.7793
5	0.0911	7.8710	0.3467	34.3420

Each operator performed AR surgery in P1 neonatal mice using conventional (*n* = 5) and modified (*n* = 5) AR method, respectively.

*AR* apical resection, *SD* standard deviation, *RSD* relative standard deviation.

### Cardiac regenerative responses after modified surgical AR compared to conventional AR in neonatal mice

We next compared cardiac regenerative responses between our modified surgical AR method and conventional AR method. Using conventional AR method, we also observed comparable heart weight between AR and sham group at 7 dpr and 21 dpr (Supplementary Fig. 2a, b). Meanwhile, AR mice had normal cardiac function and regenerated apex with no significant evidence of fibrosis (Supplementary Fig. 2c, d).

**Fig. 4:**
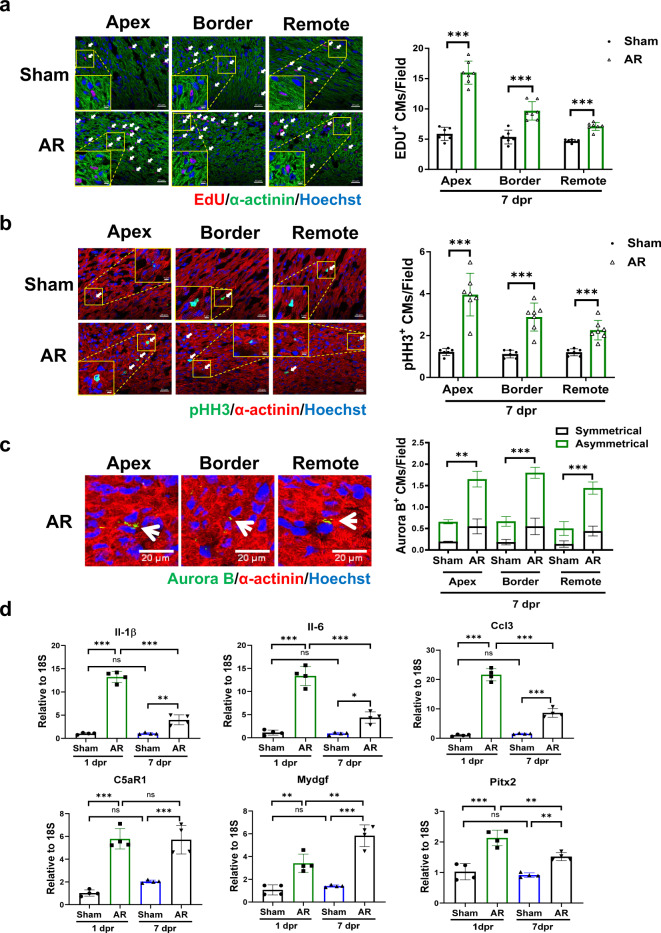
Regenerative responses after modified apical resection surgery in 1-day-old neonatal mice. Co-immunofluorescent staining for α-actinin and EdU (**a**) or phospho-histone H3 (pHH3) (**b**) in sham and apical resection (AR) hearts at 7 days post resection (7 dpr) (*n* = 6-7). Scale bar = 20 μm. A selected area with a higher magnification (Scale bar = 5 μm) for each representative image was presented. **c** Co-immunofluorescent staining for α-actinin and Aurora B at 7 dpr to evaluate Aurora B-kinase expression at the cleavage furrow between two cardiomyocytes (*n* = 4). The representative asymmetrical (in the apex and remote region) and symmetrical (in the border region) Aurora B positive cardiomyocytes were indicated by white arrows. Scale bar = 20 μm. **d** Quantitative PCR for inflammatory and immunomodulatory factors and myocardial pro-proliferative factors in sham and AR hearts at 1 dpr and 7 dpr (*n* = 4). **P < 0.01; ***P < 0.001; ns not significant. All data are expressed as means ± SD.

Conventional AR surgery in P1 mice induces a robust cardiomyocyte proliferative response in the whole heart^4^. In our study, cardiomyocyte proliferation and cytokinesis markers were assessed by colocalization of EdU, pHH3, or Aurora B-kinase with α-actinin positive cardiomyocytes. Consistent with previous reports, increase of EDU, pHH3, and Aurora B-kinase positive cardiomyocytes was observed in the apex, apical border zones, and remote zones of resected hearts after conventional AR at 7 dpr (Supplementary Fig. 3). We then examined whether modified AR method was able to stimulate cardiomyocyte proliferative response in neonatal mice. Similarly, we observed a significant increase of EdU, pHH3, and Aurora B-kinase positive cardiomyocytes in the apex, apical border zones, and remote zones of resected hearts after modified AR at 7 dpr (Fig. 4a–c).

Previous studies demonstrated marked induction of inflammatory and immune responses during early cardiac regeneration after AR surgery, which has been suggested to play an important role in promoting cardiomyocyte proliferation and regenerative response^4,14^. Thus, we determined these reported factors in AR heart tissues at 1 dpr and 7 dpr after modified AR surgery. Our results demonstrated obvious upregulation of inflammatory and immunomodulatory factors (Il-6, Il-1β, Ccl3, and C5aR1) at 1 dpr; these factors were still upregulated at 7 dpr in the AR group compared to sham group (Fig. 4d). Meanwhile, we observed upregulation of myocardial pro-proliferative factors including Mydgf and Pitx2 at mRNA level in AR heart tissues with modified AR method at 1 dpr and 7 dpr (Fig. 4d).

Taken together, these findings of our modified AR model are consistent with previous observations that cardiac regeneration after AR surgery involves an inflammatory response and widespread proliferation of cardiomyocytes. The cardiac regenerative responses in modified AR model are comparable to those in conventional AR model.

### Modified AR method induces cardiac structural and functional recovery in neonatal rats

Next, we sought to establish and evaluate the modified AR method in neonatal rats. We first prepared a modified 200 μL pipette tip with its tip cut to 1.46 mm in internal diameter (Supplementary Fig. 4a). The weight of resected apex was proven to be 12% of whole heart weight by 3 independent experiments (Supplementary Table 2). At 56 dpr, we found that both heart weight and body weight were lower in AR group than those in sham group (Supplementary Fig. 4b). Although 12% AR rats had normal cardiac function at 56 dpr (Supplementary Fig. 4c), HE staining and Masson’s trichrome staining showed incomplete regeneration and significant fibrosis in the apex (Supplementary Fig. 4d). We then tried to reduce the resection weight using a modified 200 μL pipette tip with its tip cut to 0.72 mm in internal diameter (Supplementary Fig. 5a), and confirmed a resection of 7% of the whole heart weight by 3 independent experiments using this AR approach (Supplementary Table 3). Although the heart weight and cardiac function of the 7% AR group didn’t show significant difference compared to sham group at 42 dpr (Supplementary Fig. 5b, c), 7% AR rats still failed to regenerate their myocardium and had fibrotic tissues in the apex (Supplementary Fig. 5d). Thus, 12% and 7% AR did not lead to complete apical recovery in neonatal rats.

**Fig. 5:**
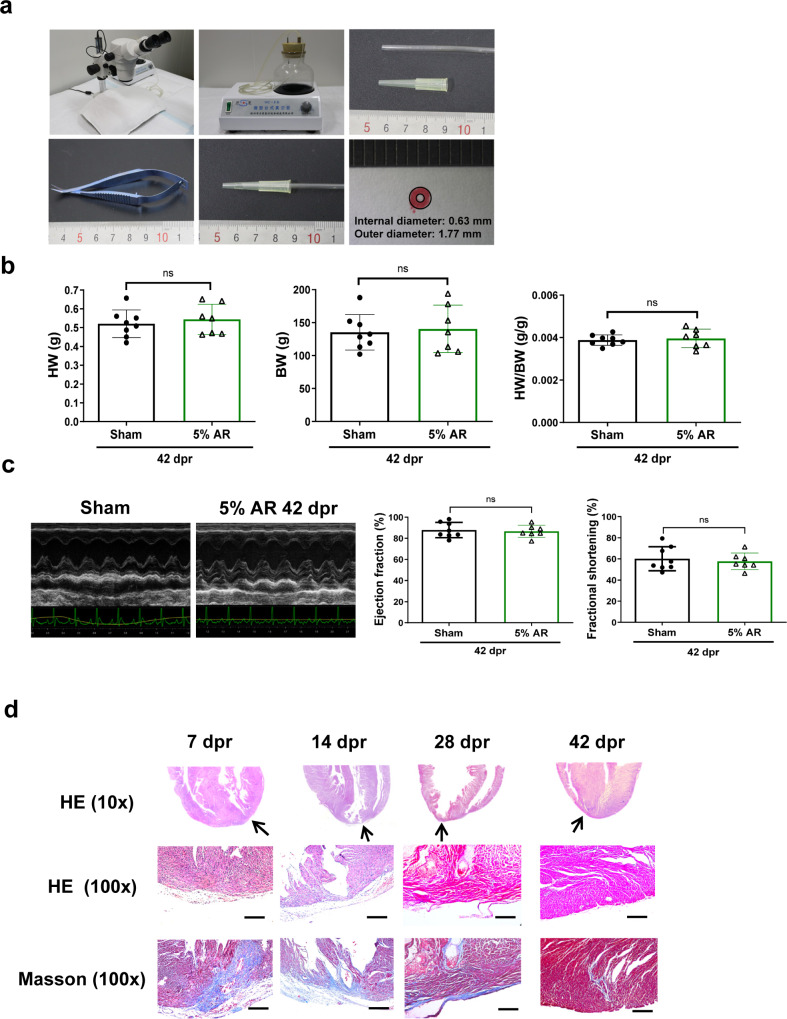
Cardiac structural and functional recovery after 5% modified apical resection surgery in 1-day-old neonatal rats. **a** A stereoscope, a desktop vacuum pump, and a 200 μL pipette tip with its tip cut to 0.63 mm in internal diameter using a sharp blade, were prepared for modified apical resection (AR) method. **b** Heart weight (HW), body weight (BW), and heart weight/body weight ratio (HW/BW) at 42 days post resection (dpr) (*n* = 7-8). **c** Echocardiography for left ventricle ejection fraction and fractional shortening at 42 dpr (*n* = 7-8). **d** Representative images for hematoxylin-eosin (HE) and Masson’s trichrome staining for AR hearts at 7, 14, 28, and 42 dpr. Scale bar = 200 μm. ns not significant. All data are expressed as means ± SD.

Given the incomplete recovery after 12% and 7% AR in neonatal rats, we decided to further reduce the resection weight to 5% of whole heart weight. We prepared a modified 200 μL pipette tip with its tip cut to 0.63 mm in internal diameter, which was connected to a vacuum pump working at 0.06 ± 0.005 MPa (Fig. 5a). The tip was kept in touch with LV apex for 12 seconds and the apex was then carefully resected by a single incision adjacent to the pipette tip. The weight of the resected apical myocardium was proven to be 5% of whole heart weight by 3 independent experiments (Supplementary Table 4). An online video (Supplementary Video 1) is available for demonstration of the modified AR model in P1 neonatal rats.

Restoration of neonatal rat heart weight was observed from 7 dpr to 42 dpr, showing that there was no difference in heart weight between AR group and sham group (Supplementary Fig. 6a and Fig. 5b). Echocardiography showed that the regenerated rat hearts had normal cardiac function similar to sham hearts at 14 dpr, 28 dpr, and 42 dpr (Supplementary Fig. 6b and Fig. 5c). Histology analysis demonstrated progressive myocardial regeneration and replacement of fibrosis from 7 dpr to 42 dpr, and restoration of the LV apex and minimum fibrosis were observed at 42 dpr (Fig. 5d). These data suggest structural and functional recovery of apical heart tissue after 5% AR in P1 neonatal rats.

**Fig. 6:**
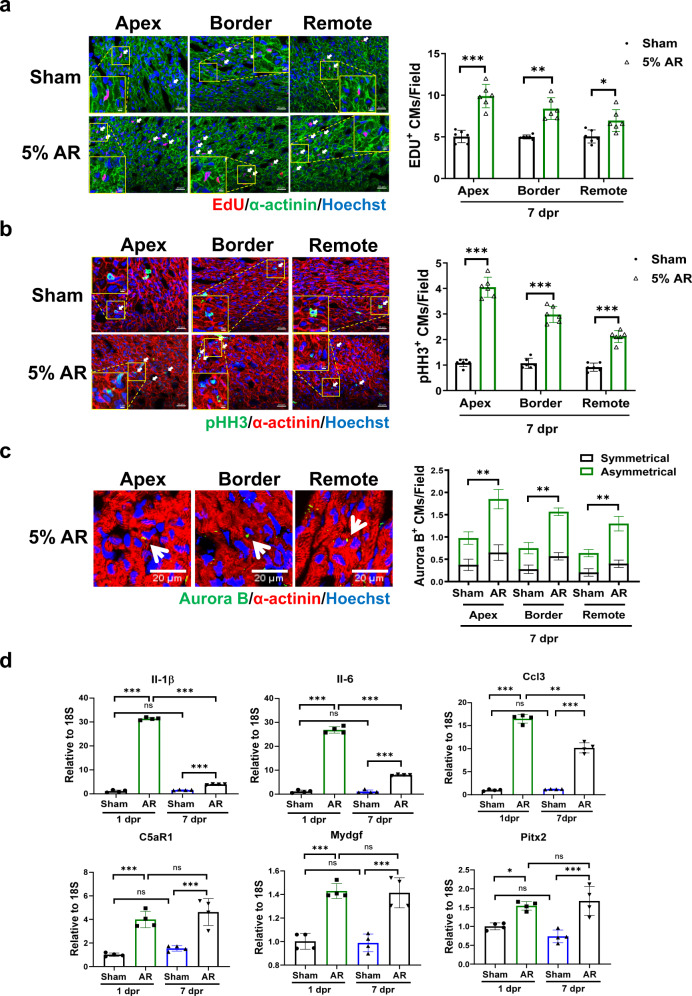
Regenerative responses after 5% modified apical resection surgery in 1-day-old neonatal rats. Co-immunofluorescent staining for α-actinin and EdU (**a**) or phospho-histone H3 (pHH3) (**b**) in sham and apical resection (AR) hearts at 7 days post resection (7 dpr) (*n* = 6). Scale bar = 20 μm. A selected area with a higher magnification (Scale bar = 5 μm) for each representative image was presented. **c** Co-immunofluorescent staining for α-actinin and Aurora B at 7 dpr to evaluate Aurora B-kinase expression at the cleavage furrow between two cardiomyocytes (*n* = 4). The representative asymmetrical Aurora B positive cardiomyocytes were indicated by white arrows. Scale bar = 20 μm. **d** Quantitative PCR for inflammatory and immunomodulatory factors and myocardial pro-proliferative factors in sham and AR hearts at 1 dpr and 7 dpr (*n* = 4). *P < 0.05; **P < 0.01; ***P < 0.001; ns not significant. All data are expressed as means ± SD.

Next, we evaluated cardiomyocyte proliferation as well as markers of inflammatory and proliferative responses after AR. We found a significant increase of EDU, pHH3, and Aurora B-kinase positive cardiomyocytes in the apex, apical border zones, and remote zones of resected hearts at 7 dpr (Fig. 6a–c). Similar to results in apically resected neonatal mice, we observed acute upregulation of Il-6, Il-1β, Ccl3, and C5aR1 at 1 dpr and 7 dpr, indicating an inflammatory response in the early stage of neonatal rat cardiac regeneration (Fig. 6d). Moreover, we observed upregulated Mydgf and Pitx2 and reduced YAP phosphorylation at Ser127 in AR heart tissues at 1 dpr and 7 dpr (Fig. 6d and Supplementary Fig. 7), suggesting a myocardial proliferative response in P1 neonatal rat after modified AR surgery. Taken together, these findings indicate that the regeneration of neonatal rat hearts involves an inflammatory response and widespread proliferation of cardiomyocytes.

### Cardiac function under hemodynamic stress is comparable between AR and sham rodents

To determine whether cardiac function under hemodynamic stress could be comparable or altered in AR group compared to sham group, we treated mice and rats with intraperitoneal injection of phenylephrine and measured cardiac function, respectively. In P1 AR-operated mice, basic cardiac function was comparable to sham mice at 42 dpr (Supplementary Fig. 8a). After a single injection of 300 μg/kg phenylephrine in mice, tail cuff blood pressure was measured to verify that SBP was increased over 40%, and there was no difference in the increase of SBP, DBP, and MBP between AR group and sham group (Supplementary Fig. 8b). In mice with SBP increased over 40%, cardiac function was found to be decreased but to a comparable degree in AR group and sham group (Supplementary Fig. 8c). These data suggest that both sham and AR mice have comparable cardiac function under hemodynamic stress.

**Fig. 7:**
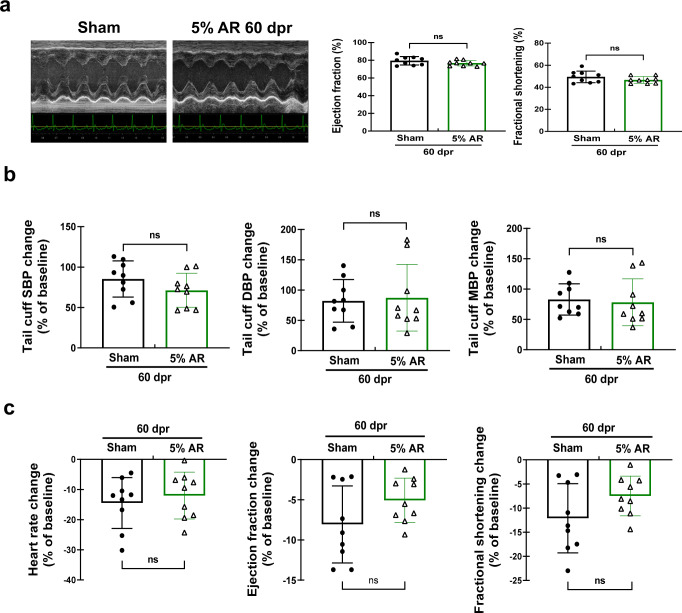
Long-term cardiac function under hemodynamic stress after 5% modified apical resection surgery in 1-day-old neonatal rats. **a** Echocardiography for left ventricle ejection fraction and fractional shortening at baseline before phenylephrine injection in rats at 60 days post resection (dpr) (*n* = 9). **b** Tail cuff blood pressure measurements including systolic blood pressure (SBP), diastolic blood pressure (DBP), and mean blood pressure (MBP) (*n* = 9). Data were presented as change from baseline (%) once SBP increased over 40%. **c** Echocardiography for heart rate, left ventricle ejection fraction, and fractional shortening when SBP increased over 40% from baseline after phenylephrine injection (*n* = 9). Data were presented as change from baseline (%). ns not significant. All data are expressed as means ± SD.

Next, we also determined cardiac function of AR rats *versus* sham rats under hemodynamic stress through intraperitoneal injection of 25 mg/kg phenylephrine when they grew to adults at 60 dpr. Baseline cardiac function before injection was measured at 60 dpr, showing no difference in LV systolic function between sham and AR rats (Fig. 7a). Then tail cuff blood pressure was measured at baseline and every 2 minutes after injection, and cardiac function was measured once SBP increased over 40% from baseline. For rats whose SBP failed to increase over 40% after 5 min of injection, another injection of 12.5 mg/kg phenylephrine was given to them. Our results showed that phenylephrine induced comparable increases of SBP, DBP, and MBP in AR and sham rats (Fig. 7b). Echocardiography results showed that the reduced percentage of heart rate as well as LV ejection fraction (EF) and fractional shortening (FS) from baseline after phenylephrine injection was also comparable between AR and sham rats (Fig. 7c). These results suggest that there is no alteration of cardiac function under hemodynamic stress in 5% AR rats compared to sham rats. Collectively, we observed long-term functional recovery of neonatal rodent hearts after modified AR surgery.

### Cardiac regenerative capacity in neonatal rats is lost from 7 days post birth

To further compare cardiac regenerative capacity at different postnatal period, we additionally performed AR surgery in 3-day-old (P3) and 7-day-old (P7) rats, respectively. We observed that P3 rats with AR surgery had comparable heart weight, preserved cardiac function, and regenerated apex compared to sham rats at 42 dpr (Supplementary Fig. 9). Meanwhile, P3 rats had increased EDU, pHH3, and Aurora B-kinase positive cardiomyocytes in the apex, apical border zones, and remote zones of resected hearts at 7 dpr compared to sham rats (Supplementary Fig. 10). These data demonstrate that P3 rats can also achieve structural and functional recovery of the heart after AR surgery with cardiac regeneration.

However, we found that P7 rats were no more resistant to AR surgery using the same modified 200 μL pipette tip cut to 0.63 mm in internal diameter. Thus, a smaller proportion of the apex was resected in P7 rats using a modified 200 μL pipette tip with its tip cut to 0.57 mm in internal diameter (Supplementary Fig. 11a). Even in this way, we still found 50% mortality among apical resected P7 rats on the same day or on the next day of AR surgery. For those survived rats at 42 dpr, although P7 AR rats had comparable heart weight in comparison to sham rats (Supplementary Fig. 11b), they had slight but significant reduction of cardiac function and obvious fibrotic tissues around the apex (Supplementary Fig. 11c, d). Moreover, hardly no evidence of cardiomyocyte proliferative response was detected in the apex zone at 7 dpr (Supplementary Fig. 12). Thus, similar to previous evidence showing that the mouse heart loses regenerative capacity within the first week of postnatal life^4^, our results indicate that cardiac regenerative capacity in neonatal rats is also lost from 7 days post birth.

## Discussion

Rodent AR models are widely used to investigate regeneration of the mammalian heart. In the present study, we developed a modified AR method by using a modified pipette tip to accurately control the amount of resected apical myocardium. By comparing the modified and conventional AR methods in P1 neonatal mice, similar cardiac structural and functional recovery and cardiac regenerative responses were observed in both methods. Higher reproducibility and smaller variation of resected LV apical myocardium was achieved by using the modified AR method. Moreover, we established 5% AR of whole heart weight in P1 neonatal rats, and provided evidence that cardiac regeneration and recovery can be replicated in rats using 5% AR model.

Conventional AR method was accomplished by resecting LV apex until the exposure of LV chamber which has been used as an anatomic landmark for limited resection to ensure reproducibility of AR^3^. Despite a large majority of studies reported structural and functional recovery of hearts, some groups failed to observe complete LV apex regeneration and repair after AR surgery^15^. It is difficult to compare the resected weight or area of LV apical myocardium since some groups used the resected weight and some others used the resected area as control of AR model in neonatal mice^4,5,7,9^. Moreover, using the conventional AR method, gradually resecting the LV apex may require several incisions which increases the risk for variation of the size of resected LV apex tissue. In the present study, we prepared a modified 10 μL pipette tip by cutting its tip to 0.48 mm in internal diameter, which was then connected to a vacuum pump working at 0.06 ± 0.005 MPa for nearly but no more than 12 s. We achieved 15% AR of whole heart weight in neonatal P1 mice by a single incision adjacent to the pipette tip. A structural apical recovery with minimal evidence of fibrosis and normal cardiac function were observed in AR mice at 21 dpr, which was consistent with previous studies using a conventional AR method^4^. Thus, the modified 15% AR method in P1 neonatal mice achieved cardiac structural and functional recovery with some advantages. With the modified pipette tip connected to a vacuum pump, it is much easier to accurately and uniformly resect part of the LV apex. Meanwhile, a single incision of LV apex adjacent to the pipette tip in the present method is more practical to operate the surgery procedure. Moreover, the needed instruments and materials are easy to obtain and cost-effective, which ensure easy application of this modified AR method by any laboratories.

Surgical variation of animal model may lead to different degrees of injury which influences the outcomes of pathological processes. After confirming the success of cardiac structural and functional recovery in our modified AR model in P1 neonatal mice, we further tried to evaluate the homogeneity and reproducibility of this modified method in comparison to conventional AR method. After training, all operators can perform the AR surgery in P1 neonatal mice within 10 min and achieved an exposure of LV chamber with a survival rate over 80%, indicating that all operators mastered the conventional and modified AR methods to a same degree. In comparison to the modified method, we first observed that the 5 operators involved in our study practiced more mice to learn and achieve a surgical AR procedure time within 10 min using the conventional method. We further observed smaller variation of resected LV apex using the modified AR method for each operator, and demonstrated a significant lower RSD of resected LV apex for 5 operators using the modified AR method compared to the conventional AR method. Collectively, these data suggest that the modified AR method is easy to learn and has higher homogeneity and reproducibility than conventional AR method.

Cardiac regenerative responses in the early phase after AR surgery play important roles in promoting cardiac regeneration and recovery^7,14^. In a cross-species transcriptomic screen in axolotl, neonatal mice, and zebrafish AR models, a conserved upregulation of genes at 12 to 48 hours post resection were found to be enriched for gene ontology analysis such as inflammatory response, immune system process and regulation, immune and defense response, and cell proliferation^14^. Based on this screening, complement 5a receptor 1 (C5aR1) was revealed to be significantly induced after AR, which further contributed to cardiomyocyte proliferative response and played an essential role for early cardiac regeneration after AR^14^. In the present study, we observed a significant increase of EdU, pHH3, and Aurora B-kinase positive cardiomyocytes in the apex, apical border zones, and remote zones of resected hearts at 7 dpr in the modified AR model, which was consistent to the robust cardiomyocyte proliferative response in the conventional AR model as previously reported^4^. Meanwhile, we observed obvious upregulation of inflammatory and immunomodulatory factors (Il-6, Il-1β, Ccl3, and C5aR1) in the modified AR mouse model at 1 dpr and 7 dpr, which was consistent to previous reports showing acute inflammatory responses after AR^4,14^. By determining some previously reported myocardial pro-proliferative factors^5,16^, we observed upregulation of Mydgf and Pitx2 in resected heart tissues with modified AR method at 1 dpr and 7 dpr, further validating molecular changes of myocardial pro-proliferative factors in this modified AR mouse model.

Despite a validation of cardiac regeneration after AR in neonatal mice, whether and how cardiac regenerative capacity could be replicated in neonatal rats has previously been unclear. A previous study performing AR with 16–18% resected apical area in P1 neonatal rats as estimated by magnetic resonance imaging (MRI), reported an incomplete cardiac recovery accompanied with myocardial hypoperfusion and cardiac fibrosis at 60 dpr^13^. Here, we hypothesized that the resected part of the heart may be too large to accomplish cardiac structural and functional recovery post resection in rats. By gradually reducing the internal diameter of the modified 200 μL pipette tip to 0.63 mm, we finally succeeded in establishing 5% AR of whole heart weight in P1 neonatal rats which led to complete regeneration of LV apex and full preservation of cardiac function at 42 dpr. Similar to the AR mouse model and previously reported cardiac regeneration studies^4,5,14,16–18^, an obvious cardiomyocyte proliferative response in the whole heart and marked upregulation of inflammatory and immunomodulatory factors (Il-6, Il-1β, Ccl3, and C5aR1) and regulation of myocardial pro-proliferative factors (upregulation of Mydgf and Pitx2 and reduced phosphorylation of YAP at Ser127) were observed in resected hearts at 1 dpr and 7 dpr. Moreover, we demonstrated that similar to AR-operated neonatal mice, AR-operated neonatal rats displayed comparable reduced heart rate and LV systolic function upon phenylephrine-induced hemodynamic stress when compared to sham rats even at 60 dpr, suggesting a long-term functional recovery of neonatal rat hearts after 5% AR surgery. Thus, using this modified AR method, we established 5% AR neonatal rat model which will provide a necessary basis for subsequent research on heart regeneration in genetically modified neonatal rats.

Here we further observed that both P1 and P3 neonatal rats have cardiac regenerative capacity and achieve functional and structural recovery of the heart after AR. However, this cardiac regenerative potential is lost in P7 rats, since AR caused reduced cardiac function and fibrotic tissue growth around the resected apex at 42 dpr. Moreover, hardly no evidence of cardiomyocyte proliferative response was observed around the resected apex at 7 dpr. This may be closely related to a rapid transition of cardiac myocytes from hyperplasia to hypertrophy within one week after birth^4,19^.

In conclusion, we develop a modified easy-to-control and easy-to-perform AR method by using a modified pipette tip connected to a working vacuum pump to accurately control the resection part of LV apex in rodents. This modified AR method with high homogeneity and reproducibility induces cardiac structural and functional recovery in neonatal rodents which can be practically applied by researchers after quick learning. A 5% AR neonatal rat model is proven to be practical to induce structural and functional cardiac regeneration with minimal fibrosis of LV apex. We think that the modified AR method in rodents will prove to be useful for structural, functional, and molecular investigations of cardiac regeneration and repair in mammals.

## Methods

### Animals and ethic statements

Eight-week-old C57BL/6J mice and Sprague-Dawley rats were purchased from Cavens Lab Animal (Changzhou, China) and maintained in the specific-pathogen-free (SPF) laboratory animal facility of Shanghai University (Shanghai, China). Their offspring was used to establish AR models in neonatal rodents. All animal experiments were conducted in concordance with the Guidelines for the Care and Use of Laboratory Animals for biomedical research published by the National Institutes of Health (No. 85-23, revised 1996) and approved by the Committee for the Ethics of Animal Experiments of Shanghai University.

### Modified and conventional AR models of neonatal mouse heart

The modified AR method was conducted and evaluated in P1 neonatal C57BL/6J mice. For an AR of 15% weight of the whole heart, a modified 10 μL pipette tip was prepared by cutting its tip to 0.48 mm in internal diameter using a sharp blade. A formula was given to calculate the distance cutting from the pipette tip (Supplementary Fig. 13). The operators could calculate the distance cutting from the pipette tip after measuring the parameters of their commonly used pipette tip or according to the standard size of the pipette tip provided by manufacturers. Before the beginning of the surgical procedure, all P1 neonatal mice were removed from their mother into a new clean cage with bedding materials. The neonatal mouse was wrapped with gauze to avoid frostbite and then put on ice for 3–5 min. Under hypothermia anesthesia, temporary apnea and asystole were achieved. No analgesic drug was used during the surgery. The neonatal mouse was then transferred to a surgical area under a stereoscope and placed in the supine position immobilized with medical adhesive tape. A transverse skin incision was performed below the bilateral fossa axillaris using blunt scissors. After blunt dissection at the fourth intercostal space, a 0.4 to 0.6-cm-long incision was gently performed to enter the chest cavity and visualize the heart. The modified 10 μL pipette tip, with its tip cut to 0.48 mm in internal diameter, was connected to a vacuum pump working at 0.06 ± 0.005 MPa and gently kept in touch with LV apex for 12 s. The LV apex was carefully resected by a single incision adjacent to the pipette tip using iridectomy scissors. Of note, a pipetting time over 12 s was avoided as it may cause a larger resection area and subsequently incomplete and fibrotic repair of the heart. After AR surgery was completed, the ribs, muscles and then the skin were sutured using 7-0 surgical sutures. The blood was gently cleaned from the pups and the chest was disinfected with iodine. Animals were quickly placed on a heating pad for recovery. The complete procedure time, from anesthesia to chest closure was aimed to be below 10 min. Sham mice underwent the same surgical procedures without pipette tip suction and AR. After all animals were recovered with spontaneous breathing, they were returned to their mother’s cage. Animals were gently wiped with the bedding materials and feces of their mother’s cage to reduce the chance of cannibalization.

To evaluate the AR operation homogeneity and reproducibility, we trained 5 operators to perform both conventional and modified AR methods. Conventional AR was accomplished by gently resecting the LV apex until exposure of the LV chamber; this is commonly used as an anatomic landmark and achieves about 15% reduction of the whole heart weight in P1 neonatal mice^3,4^. To compare the AR methods, the operators resected LV apex with a single incision for both the conventional and the modified AR approach in the present study. Apart from the use of a modified pipette tip in the modified AR procedure, other techniques were the same between the two methods as described above. A surgical time from anesthesia to chest closure within 10 min and a survival rate after AR over 80% were required and indicative of mastering the AR method.

Quickly after AR surgery, resected and intact hearts were rinsed briefly with phosphate buffered saline (PBS) and weighed, in order to validate the 15% AR of whole heart weight. At 7 dpr and 21 dpr, the heart weight (HW), body weight (BW), and heart weight/body weight ratio (HW/BW) were measured. Echocardiography was performed at 21 dpr. Heart tissues were harvested for histological analysis (7 dpr and 21 dpr), immunofluorescent stainings for cardiomyocyte proliferative markers (7 dpr), and molecular analysis for markers of inflammatory and myocardial proliferative responses (1 dpr and 7 dpr).

### Modified AR model of neonatal rat heart

The modified AR method was conducted and evaluated in P1 neonatal Sprague-Dawley rats. A modified 200 μL pipette tip was prepared by cutting its tip to 0.63 mm in internal diameter using a sharp blade, which achieved a 5% AR of whole heart weight in P1 neonatal rats. Except for a slightly larger incision (0.6 to 0.8 cm-long) for entering the chest cavity and visualizing the heart and a modified 200 μL pipette tip with its tip cut to 0.63 mm in internal diameter, the other procedures were the same as those described for the modified AR method of neonatal mouse heart. The precise position where a pipette tip attaches to the apex is shown in Supplementary Fig. 14a, b. A pipetting time nearly but no more than 12 s did not cause damage to the apex (Supplementary Fig. 14c), while a pipetting time over 12 s risked to squeeze the apex and may cause damage to the apex (Supplementary Fig. 14d). To further confirm that the use of pipetting within 12 seconds per se did not cause damage to the heart, we also set up a group of pipette suction after thoracotomy without AR and evaluated cardiac function and histology of cardiac tissues at 7 days post operation. Compared to those P1 neonatal rats without pipette suction, a pipetting time about 12 s did not influence cardiac function or cause pathological changes of cardiac tissues at 7 days post operation (Supplementary Fig. 15). These results indicate that the use of pipette suction within 12 s does not cause damage to the heart. The surgical time from anesthesia to chest closure should be limited within 10 min, and this modified AR method in P1 neonatal rat can achieve a survival rate over 80%. As indicated in Results section, a schematic flow chart (Fig. 1) and an online video (Supplementary Video 1) are available for demonstration of the modified AR model in P1 neonatal rodents.

Quickly after AR surgery, resected and intact hearts were rinsed with PBS and weighed. The weight of resected LV apex was proven to be 5% of whole heart weight in P1 neonatal rats by 3 independent experiments. At 7, 14, 28, and 42 dpr, the HW, BW, and HW/BW ratio were measured. Echocardiography was performed every 2 weeks from 14 to 42 dpr. Heart tissues were harvested for histological analysis (7, 14, 28, and 42 dpr), immunofluorescent stainings for cardiomyocyte proliferative markers (7 dpr), and molecular analysis for markers of inflammatory and myocardial proliferative responses (1 dpr and 7 dpr).

To explore when neonatal rats lose cardiac regenerative capacity after birth, we further performed AR surgery in 3-day-old (P3) and 7-day-old (P7) rats, respectively. P3 rats underwent AR procedure using the same modified 200 μL pipette tip cut to 0.63 mm in internal diameter. While P7 rats underwent AR procedure using a modified 200 μL pipette tip cut to 0.57 mm in internal diameter for a smaller proportion of resected LV apex due to a higher mortality induced by AR surgery at day 7 after birth. Echocardiography was performed at 42 dpr, followed by heart tissue harvest and histological examinations. Cardiomyocyte cell cycle activity was determined by EdU, pHH3, or Aurora B and α-actinin co-stainings in heart tissues at 7 dpr.

### Echocardiography

Rodents were anesthetized with inhaled isoflurane, and echocardiography was performed using the Vevo 2100 Imaging System (FUJIFILM Visual Sonics, Toronto, Ontario, Canada). M-mode long-axis echocardiograms were obtained at the papillary muscle level to measure the LV ejection fraction (EF) and fractional shortening (FS) based on 3 consecutive cardiac cycles.

### Hematoxylin-Eosin staining and Masson’s trichrome staining

Whole heart tissues were fixed in 4% paraformaldehyde (PFA) and then embedded in paraffin. The 5-μm-thick cardiac longitudinal sections were stained with Hematoxylin-Eosin (Solarbio, G1120) and Masson’s trichrome staining (Solarbio, G1340) according to manufacturer’s instructions. Tissue stainings were observed and photographed under a biological microscope (Leica, DM3000). HE staining and Masson’s trichrome staining were analyzed to evaluate the recovery of LV apex and whether fibrotic tissue was present or not after AR.

### Immunofluorescent staining for myocardial proliferative markers

For EdU staining in heart tissues, rodents were intraperitoneally injected with 50 mg/kg EdU (Invitrogen, E10187) at 1 day and 3 days before sacrifice. Whole heart tissues were harvested and embedded into optimal cutting temperature compound (OCT), and longitudinally cut into 7-μm-thick frozen heart sections. Sections were fixed with 4% PFA, permeabilized with 0.5% Triton X-100, and then blocked with 5% bovine serum albumin (BSA) for 1 h at room temperature. Sections were incubated with the primary antibody for α-actinin (1:200 dilution, Sigma, A7811) at 4 °C overnight. After wash three times in PBS, sections were incubated with corresponding secondary antibody for 2 h at room temperature. EdU staining was performed using Cell-Light Apollo 567 Stain Kit (RiboBio, C10371-1) according to the manufacturer’s instructions. For pHH3 (or Aurora B) and α-actinin co-immunofluorescent staining, sections were fixed, permeabilized, and blocked as described above, and then incubated with primary antibodies for pHH3 (1:100 dilution, Abclonal, AP0840) or Aurora B (1:100 dilution, Sigma, A5102) and α-actinin (1:200 dilution, Sigma, A7811) at 4 °C overnight. After washing three times with PBS, sections were incubated with corresponding secondary antibodies for 2 h at room temperature. Nuclei were counterstained with Hoechst. Immunofluorescent staining images were viewed and taken under a confocal microscope (Zeiss, LSM710). The number of EdU, pHH3, or Aurora B positive α-actinin-labeled cardiomyocytes per field was determined in the apex, apical border zones, and remote zones of resected hearts and intact sham hearts. The symmetrical and asymmetrical Aurora B positive cardiomyocytes were determined as previously reported^20^.

### RNA extraction and reverse transcriptional-quantitative polymerase chain reaction

Total RNA was isolated from resected and intact hearts using Trizol RNAiso Plus kit (TaKaRa, 9109). RNA concentration was determined by Nanodrop 8000 (Thermo fisher), and reverse transcription of RNA was performed using RevertAid First Strand cDNA Synthesis Kit (ThermoFisher, K1622). Quantitative polymerase chain reactions were performed using SYBR Green reagent (TaKaRa, RR420A) on Roche LightCycler480 PCR System (Roche Diagnostics). Primer sequences used in this study are listed in Supplementary Table 5. 18S was used as an internal control for gene expression.

### Western blot

Total protein was extracted from resected and intact hearts using RIPA lysis buffer supplemented with Pierce protease and phosphatase inhibitor (ThermoFisher, 88668). Equal quantities of total protein were separated by SDS-PAGE gels and transferred to PVDF membranes. Membranes were blocked with 5% non-fat milk and then incubated with primary antibody for p-YAP1-S127 (1:1000 dilution, Abclonal, AP0489), followed by incubation with corresponding HRP-conjugated secondary antibody. Protein blots were developed using enhanced chemiluminescence kit. After, membranes were stripped and incubated with total-YAP1 antibody (1:1000 dilution, Proteintech, 13584-1-AP). GAPDH (1:1000 dilution, Bioworld, AP0063) was used as an internal control for protein loading. Protein band intensity was analyzed using ImageJ software. The phosphorylation level of YAP was determined by the ratio of p-YAP (S127) to total-YAP.

### Long-term cardiac function under hemodynamic stress

For comparison of long-term cardiac function under hemodynamic stress, rats were treated with selective α1-adrenoceptor agonist phenylephrine to induce a state of hemodynamic stress at 60 dpr followed by cardiac function measurement. Before phenylephrine treatment, sham and AR rats were anesthetized with inhaled isoflurane, and cardiac function was evaluated at baseline using echocardiography (Vevo 2100 Imaging System). Tail cuff blood pressure was measured using non-invasive Tail-Cuff blood pressure device (Softron BP-2010A, Beijing, China) including systolic blood pressure (SBP), dilated blood pressure (DBP), and mean blood pressure (MBP). Then rats were intraperitoneally injected with 25 mg/kg phenylephrine (Selleck, S2569), followed by measurement of tail cuff blood pressure every 2 min. For rats whose SBP failed to increase over 40% after 5 min of phenylephrine injection, another injection of 12.5 mg/kg phenylephrine was given to them. Cardiac function was measured once SBP increased over 40% from baseline. Cardiac function under hemodynamic stress was also measured in sham- and AR-operated mice at 42 dpr after a single intraperitoneal injection of 300 μg/kg phenylephrine once SBP increased over 40% from baseline.

### Statistical analysis

All data were analyzed using SPSS20.0 or GraphPad Prism8 and presented as means ± SD. An independent-sample two-tailed Student’s *t* test was used for comparisons between two groups. Two-way ANOVA test followed by Tukey *post hoc* test was performed to compare multiple groups. Relative standard deviation (RSD) analysis was performed for evaluation of reproducibility of modified and conventional AR methods. Differences were considered statistically significant with *P* < 0.05.

### Reporting summary

Further information on research design is available in the Nature Research Reporting Summary linked to this article.

## Supplementary information

Supplementary Material

Supplementary Video 1

Reporting Summary

## Data Availability

All data generated or analyzed during this study are included in this article and its supplementary information files. All relevant data are also available from the corresponding authors on reasonable request.
