# Supplementary material for: A modified apical resection model with high accuracy and reproducibility in neonatal mouse and rat hearts
Source: NPJ Regen Med. 2023 Feb 18;8:9. doi: 10.1038/s41536-023-00284-5 (PMC9938870; doi:10.1038/s41536-023-00284-5)
Supplement: Supplementary file 1 — Supplementary Material [file 41536_2023_284_MOESM1_ESM.pdf]

Supplementary Figures and Tables

Supplementary Figures and Figure Legends

Supplementary Figure 1

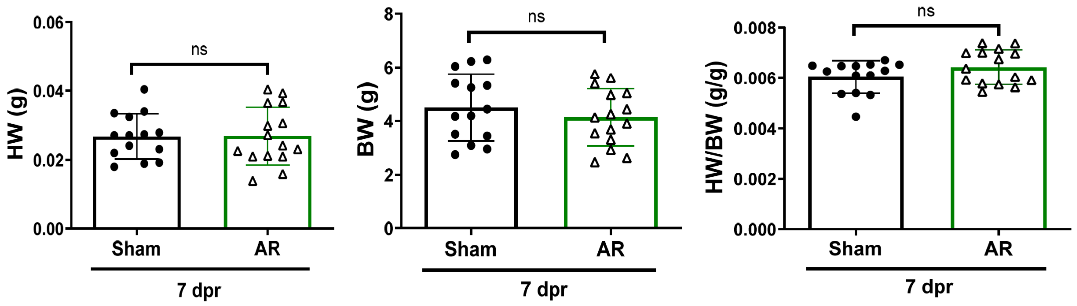

Supplementary Figure 1. Heart weight at 7 days post resection after modified apical resection surgery in 1-day-old neonatal mice. Heart weight (HW), body weight (BW), and heart weight/body weight ratio (HW/BW) at 7 days post resection (dpr) (n=14-15). ns=not significant. All data are expressed as means±SD.

Supplementary Figure 2

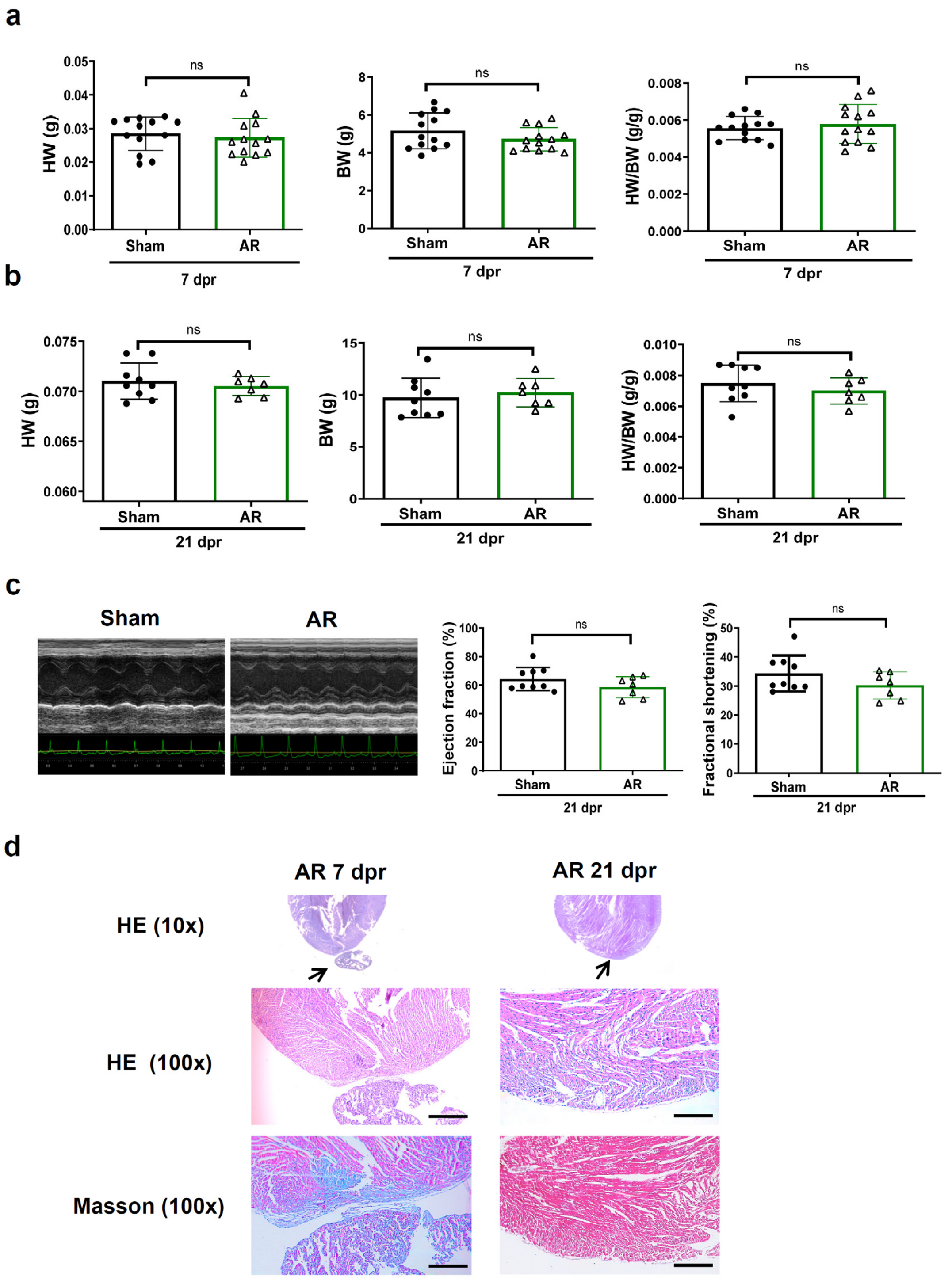

Supplementary Figure 2. Cardiac structural and functional recovery after conventional apical resection surgery in 1-day-old neonatal mice. (**a** and **b**) Heart weight (HW), body weight (BW), and heart weight/body weight ratio (HW/BW) at 7 days post resection (dpr) (**a**, n=13) and 21 dpr (**b**, n=7-9). (**c**) Echocardiography for left

ventricle ejection fraction and fractional shortening at 21 dpr (n=7-9). (**d**) Representative images for hematoxylin-eosin (HE) and Masson's trichrome staining for AR hearts at 7 dpr and 21 dpr. Scale bar=200 μm. ns=not significant. All data are expressed as means±SD.

Supplementary Figure 3

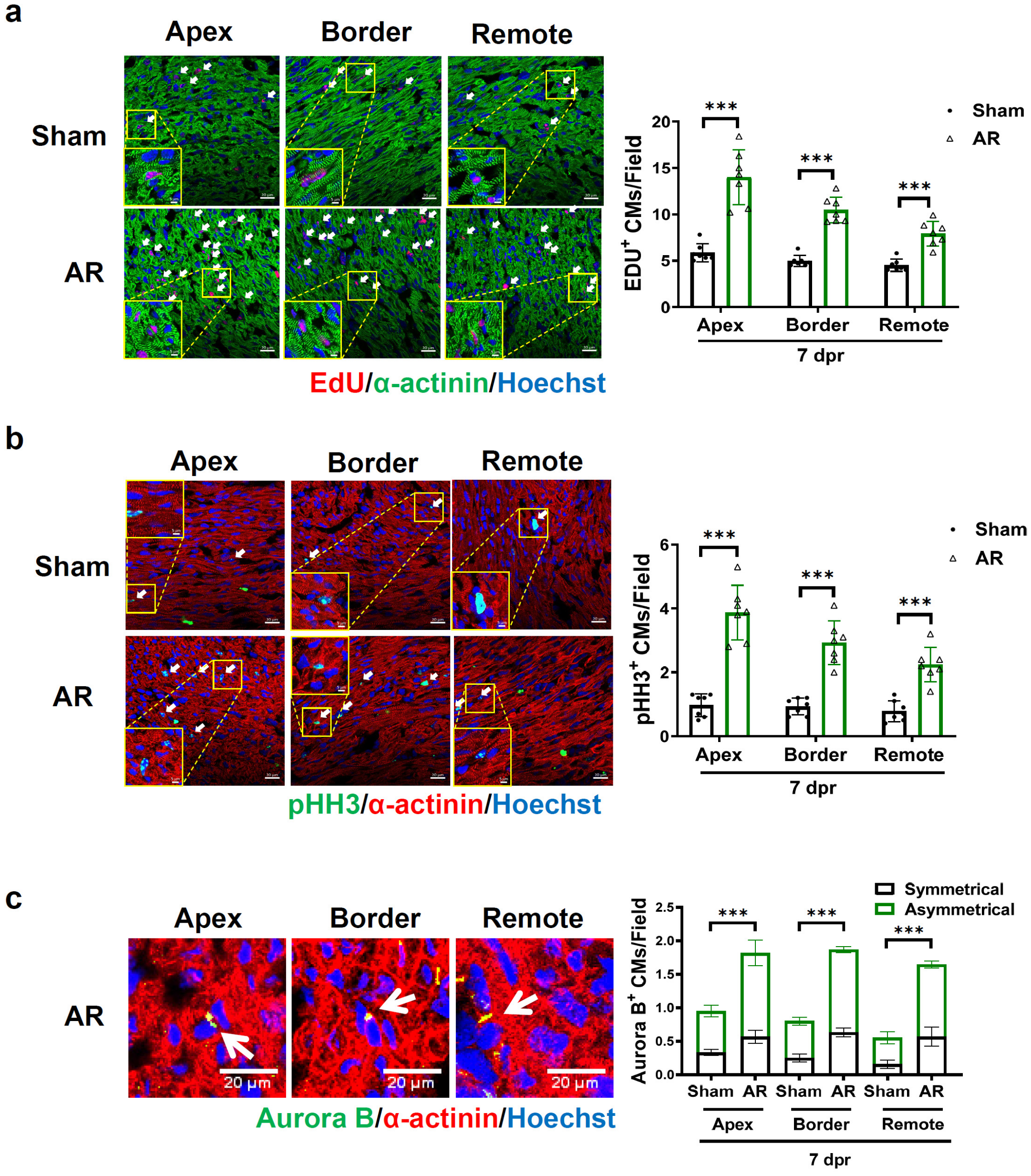

Supplementary Figure 3. Cardiomyocyte proliferation after conventional apical resection surgery in 1-day-old neonatal mice. (**a** and **b**) Co-immunofluorescent staining for α-actinin and EdU (**a**) or phospho-histone H3 (pHH3) (**b**) in sham and apical resection (AR) hearts at 7 days post resection (7 dpr) (n=7). Scale bar= 20 μm. A selected area with a higher magnification (Scale bar=5 μm) for each representative image was presented. (**c**) Co-immunofluorescent staining for α-actinin and Aurora B to evaluate Aurora B-kinase expression at the cleavage furrow between two

cardiomyocytes (n=4). The representative asymmetrical (in the apex and border region) and symmetrical (in the remote region) Aurora B positive cardiomyocytes were indicated by white arrows. Scale bar= 20 μm. ***, $P<0.001$. All data are expressed as means±SD.

Supplementary Figure 4

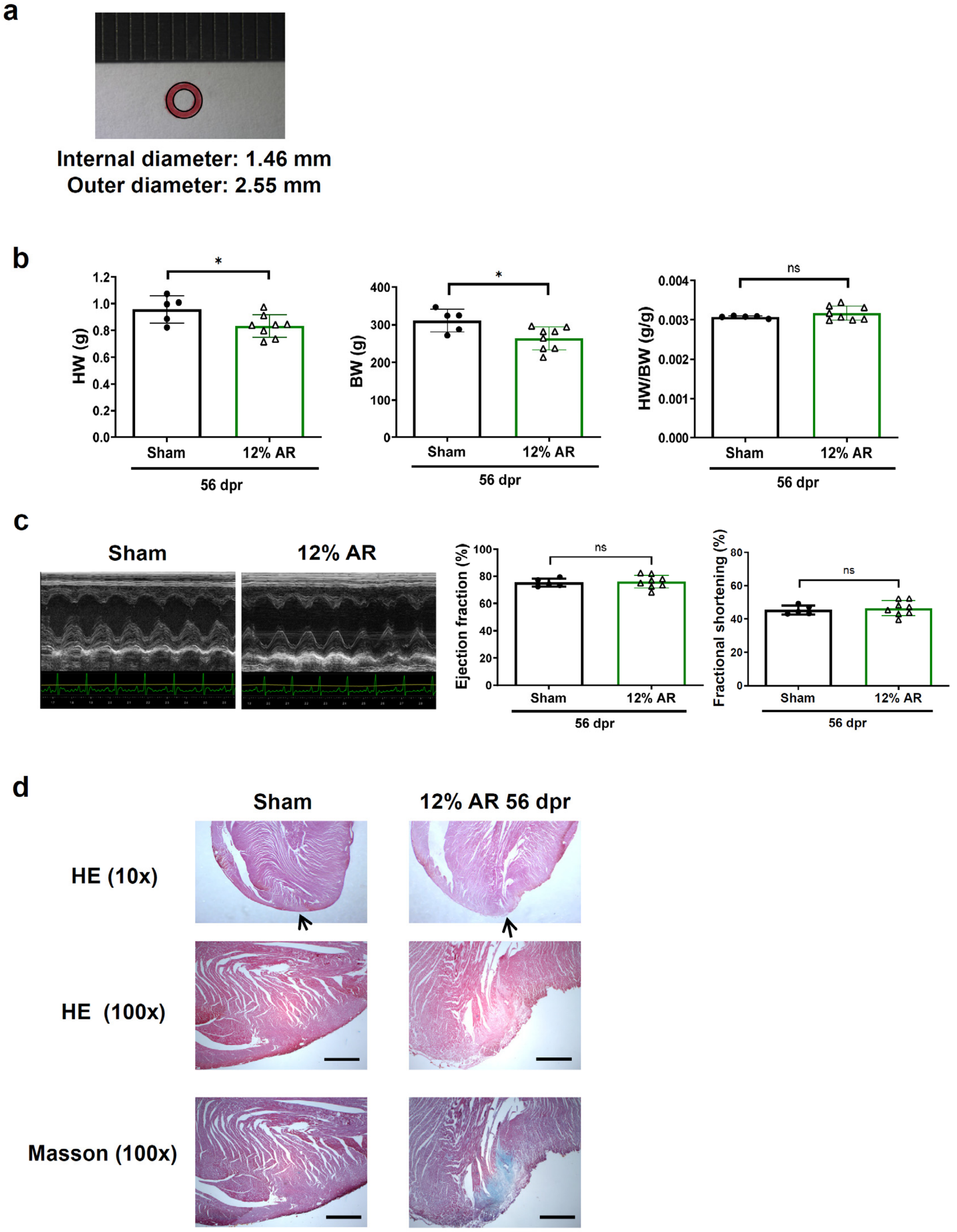

Supplementary Figure 4. Incomplete recovery of heart after 12% apical resection of whole heart weight in 1-day-old neonatal rats. (**a**) A 200 μL pipette tip with its tip cut to 1.46 mm in internal diameter was prepared to achieve 12% apical resection (AR) of whole heart weight in 1-day-old (P1) neonatal rats. (**b**) Heart weight (HW), body

weight (BW), and heart weight/body weight ratio (HW/BW) at 56 days post resection (dpr) (n=5 vs 8). (**c**) Echocardiography for left ventricle ejection fraction and fractional shortening at 56 dpr (n=5 vs 8). (**d**) Representative images for hematoxylin-eosin (HE) and Masson's trichrome staining for sham and AR hearts at 56 dpr. Scale bar=200 μm. *, P<0.05; ns=not significant. All data are expressed as means±SD.

Supplementary Figure 5

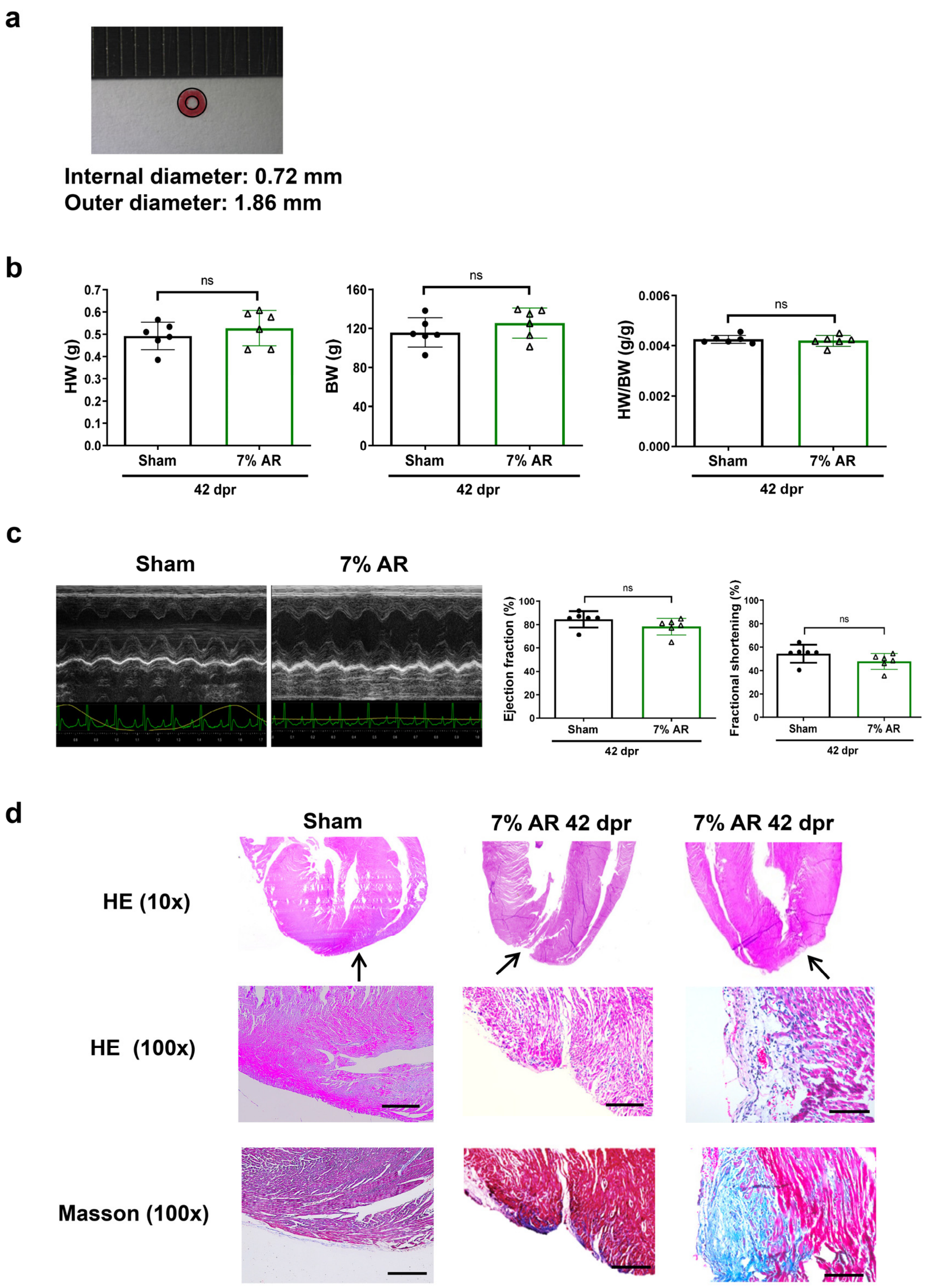

Supplementary Figure 5. Incomplete recovery of heart after 7% apical resection of whole heart weight in 1-day-old neonatal rats. (**a**) A 200 μL pipette tip with its tip cut to 0.72 mm in internal diameter was prepared to achieve 7% apical resection (AR)

of whole heart weight in 1-day-old (P1) neonatal rats. (**b**) Heart weight (HW), body weight (BW), and heart weight/body weight ratio (HW/BW) at 42 days post resection (dpr) (n=6). (**c**) Echocardiography for left ventricle ejection fraction and fractional shortening at 42 dpr (n=6). (**d**) Representative images for hematoxylin-eosin (HE) and Masson's trichrome staining for sham and AR hearts at 42 dpr. Scale bar=200 μm. ns=not significant. All data are expressed as means±SD.

Supplementary Figure 6

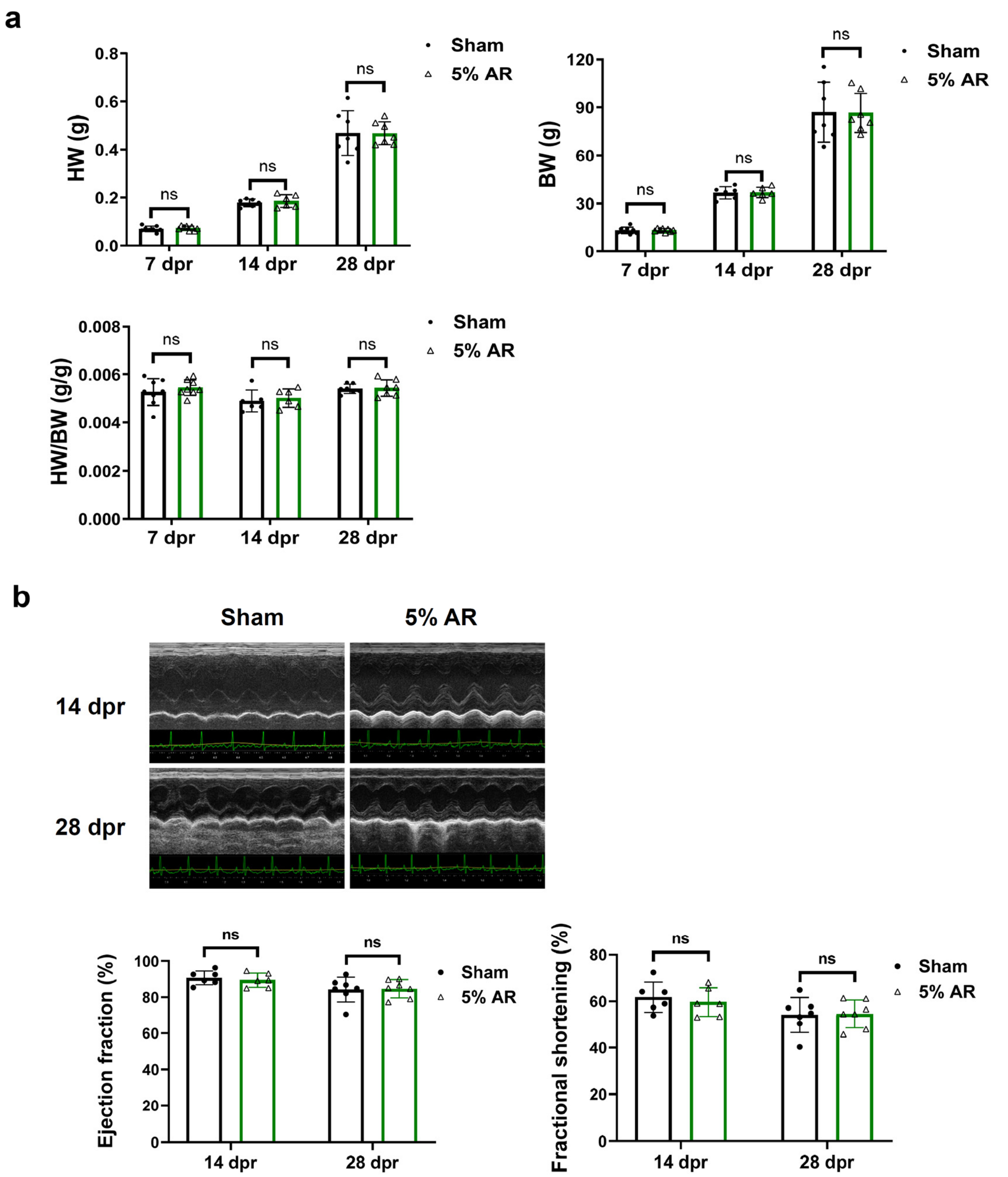

Supplementary Figure 6. Heart weight and cardiac function from 7 days to 28 days after 5% apical resection of whole heart weight in 1-day-old neonatal rats. (**a**) Heart weight (HW), body weight (BW), and heart weight/body weight ratio (HW/BW) at 7, 14, and 28 days post resection (dpr) (n=6-8). (**b**) Echocardiography for left ventricle ejection fraction and fractional shortening at 14 dpr and 28 dpr (n=6-7). ns=not significant. All data are expressed as means±SD.

Supplementary Figure 7

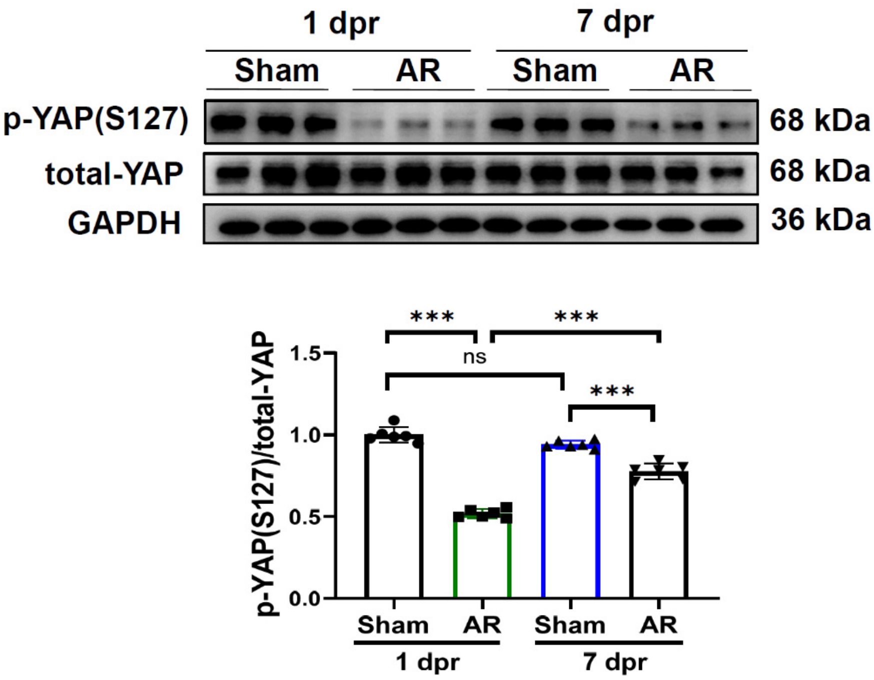

Supplementary Figure 7. Reduced YAP phosphorylation in heart tissues after 5% modified apical resection surgery in 1-day-old neonatal rats. Western blot for YAP phosphorylation at Ser127 in sham and apical resection (AR) hearts at 1 day post resection (1 dpr) and 7 days post resection (7 dpr) (n=6). ***, *P*<0.001; ns=not significant. All data are expressed as means±SD.

Supplementary Figure 8

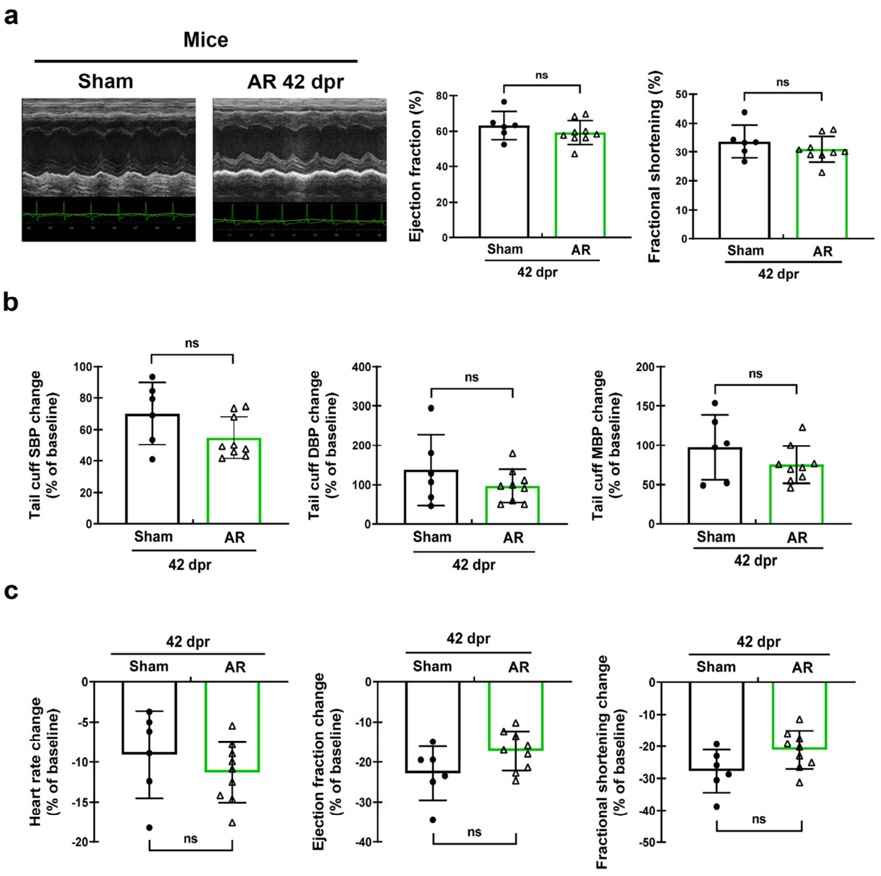

Supplementary Figure 8. Long-term cardiac function under hemodynamic stress after 15% modified apical resection surgery in 1-day-old neonatal mice. (**a**) Echocardiography for left ventricle ejection fraction and fractional shortening at baseline before phenylephrine injection in mice at 42 days post resection (dpr) (n=6 vs 9). (**b**) Tail cuff blood pressure measurements including systolic blood pressure (SBP), diastolic blood pressure (DBP), and mean blood pressure (MBP) (n=6 vs 9). Data were presented as change from baseline (%) once SBP increased over 40%. (**c**) Echocardiography for heart rate, left ventricle ejection fraction, and fractional shortening when SBP increased over 40% from baseline after phenylephrine injection (n=6 vs 9). Data were presented as change from baseline (%). ns=not significant. All data are expressed as means±SD.

Supplementary Figure 9

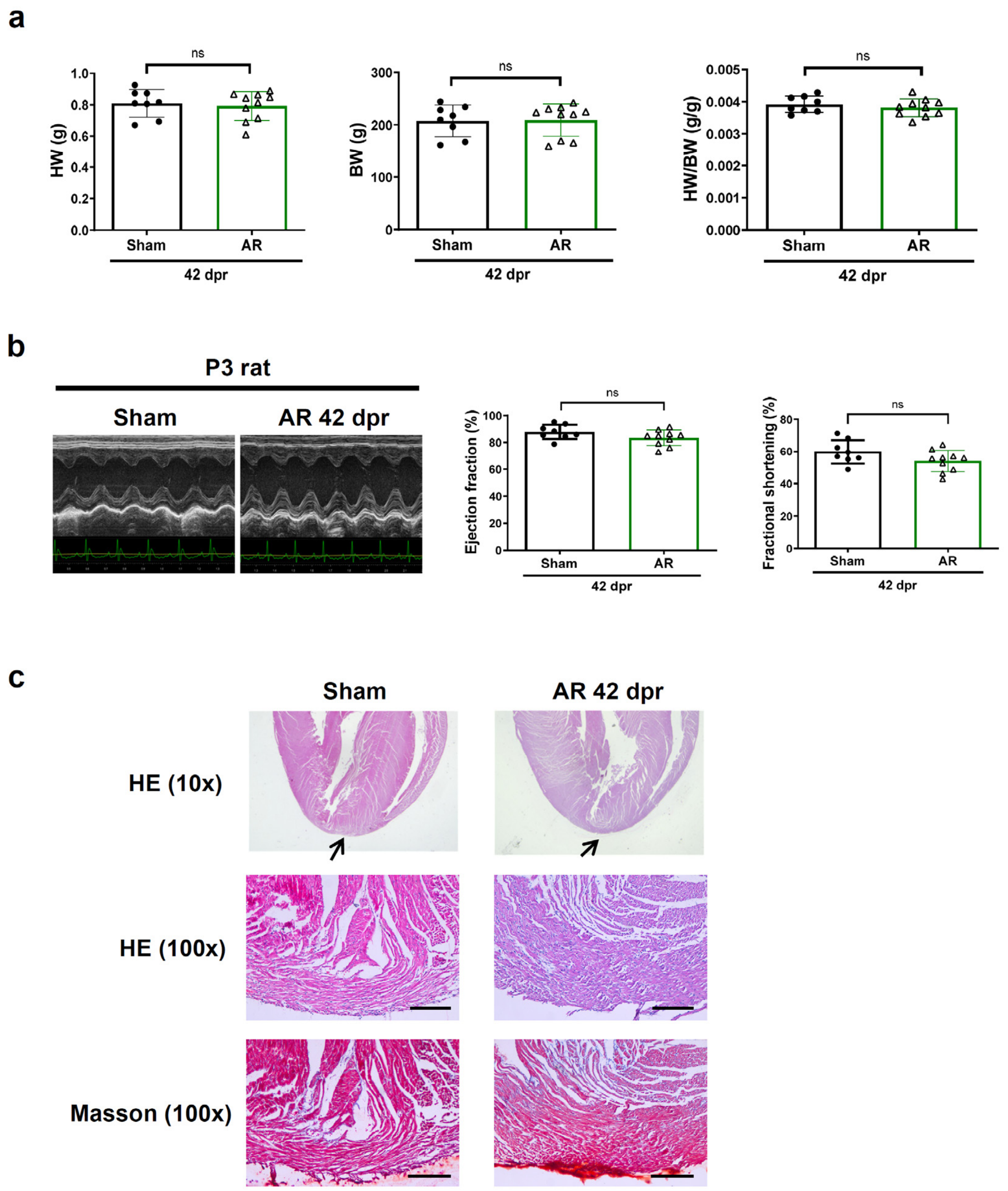

Supplementary Figure 9. Cardiac structural and functional recovery after modified apical resection surgery in 3-day-old (P3) rats. (**a**) Heart weight (HW), body weight (BW), and heart weight/body weight ratio (HW/BW) at 42 days post resection (dpr) (n=8-10). (**b**) Echocardiography for left ventricle ejection fraction and fractional shortening at 42 dpr (n=8-10). (**c**) Representative images for hematoxylin-eosin (HE) and Masson's trichrome staining for AR hearts at 42 dpr. Scale bar=200 μm. ns=not significant. All data are expressed as means±SD.

Supplementary Figure 10

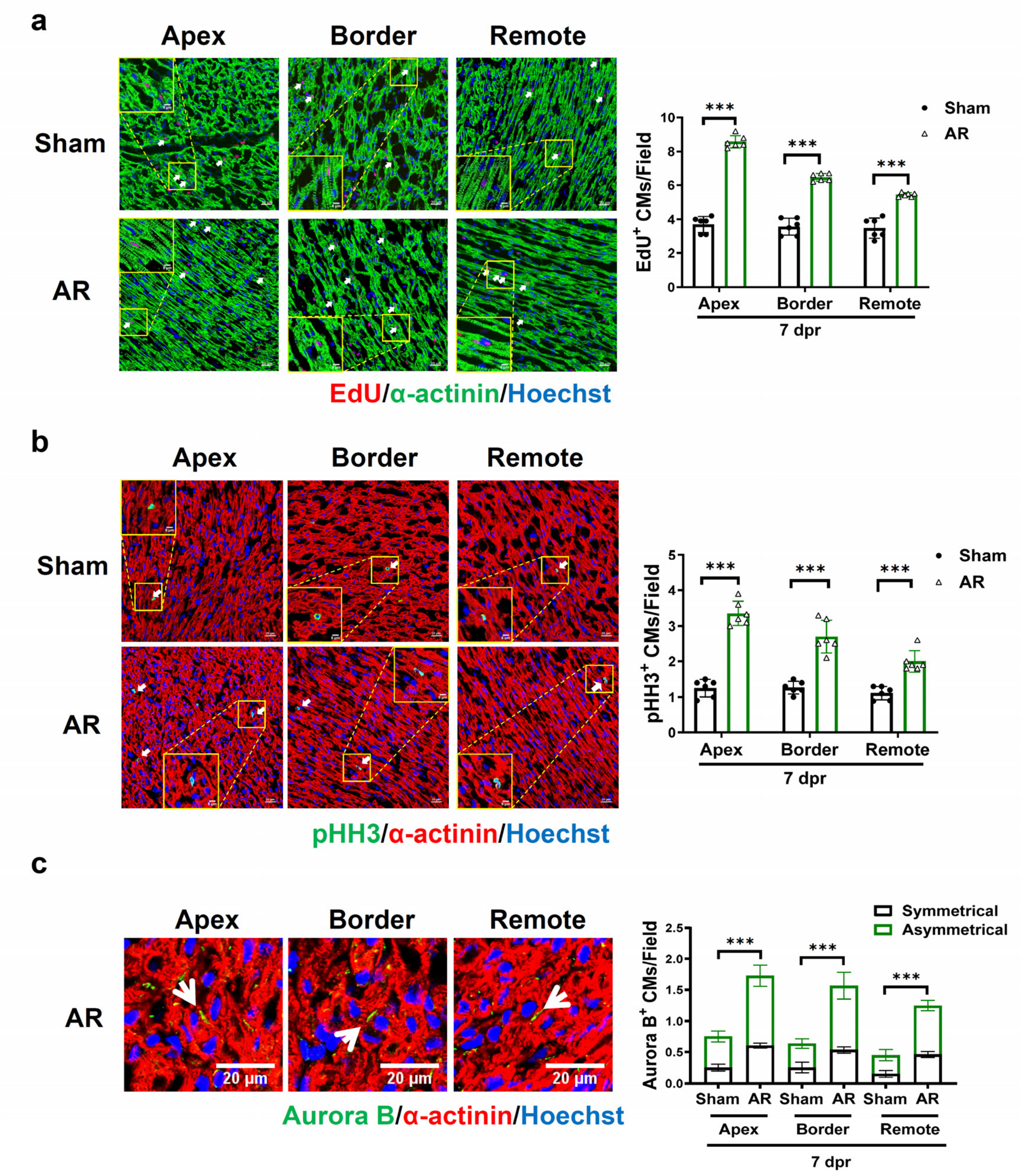

Supplementary Figure 10. Cardiomyocyte proliferation after modified apical resection surgery in 3-day-old (P3) rats. (**a** and **b**) Co-immunofluorescent staining for α-actinin and EdU (**a**) or phospho-histone H3 (pHH3) (**b**) in sham and apical resection (AR) hearts at 7 days post resection (7 dpr) (n=6). Scale bar=20 μm. A selected area with a higher magnification (Scale bar=5 μm) for each representative image was presented. (**c**) Co-immunofluorescent staining for α-actinin and Aurora B to evaluate

Aurora B-kinase expression at the cleavage furrow between two cardiomyocytes (n=6). The representative asymmetrical Aurora B positive cardiomyocytes were indicated by white arrows. Scale bar=20 μm. ***, $P<0.001$. All data are expressed as means±SD.

Supplementary Figure 11

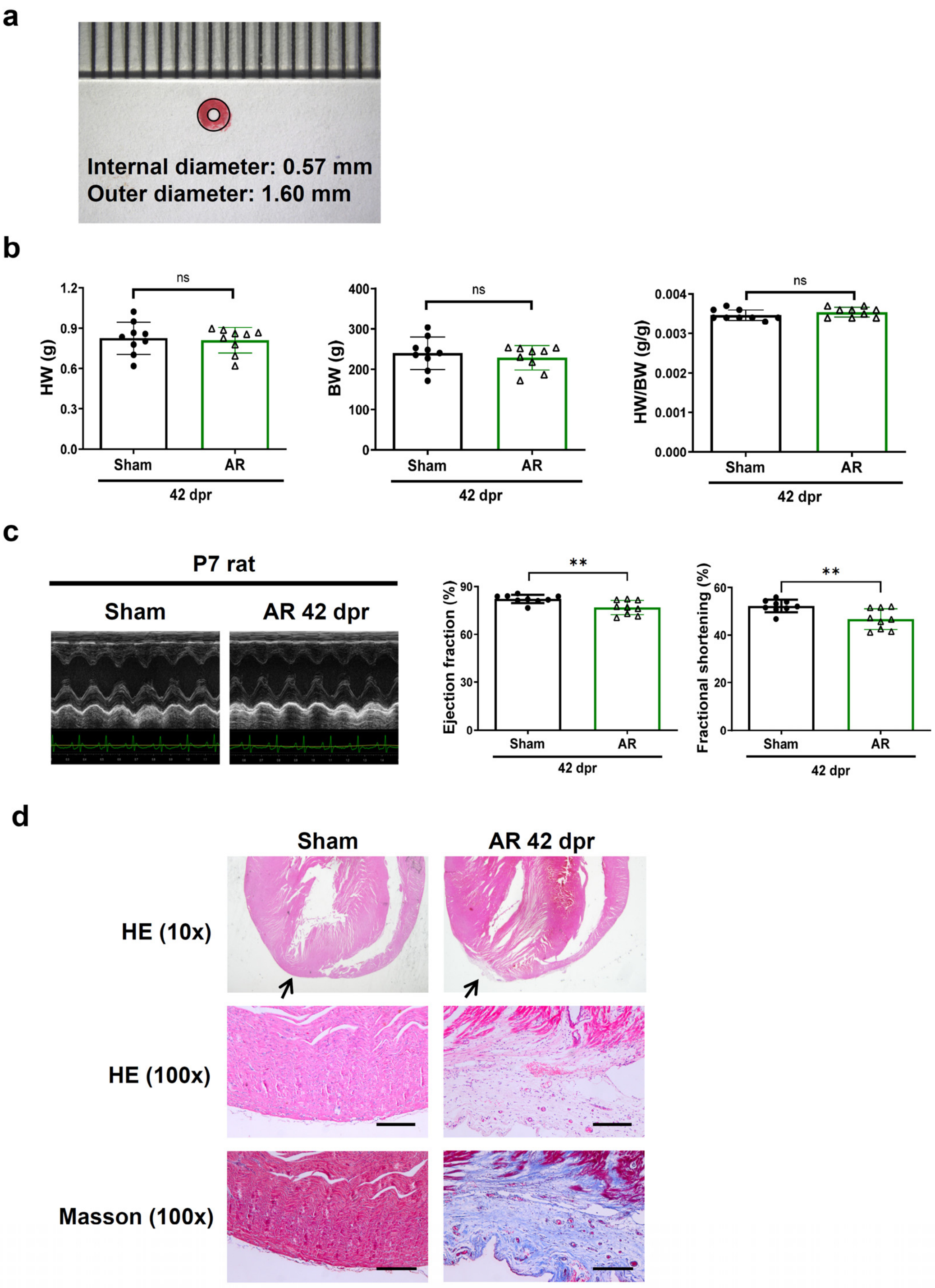

Supplementary Figure 11. Absence of cardiac structural and functional recovery after modified apical resection surgery in 7-day-old (P7) rats. (**a**) A 200 μL pipette tip with its tip cut to 0.57 mm in internal diameter was prepared to perform apical resection (AR) surgery in P7 rats. (**b**) Heart weight (HW), body weight (BW), and heart

weight/body weight ratio (HW/BW) at 42 days post resection (dpr) (n=9). (**c**) Echocardiography for left ventricle ejection fraction and fractional shortening at 42 dpr (n=9). (**d**) Representative images for hematoxylin-eosin (HE) and Masson's trichrome staining for AR hearts at 42 dpr. Scale bar=200 μm. **, $P<0.01$; ns=not significant. All data are expressed as means±SD.

Supplementary Figure 12

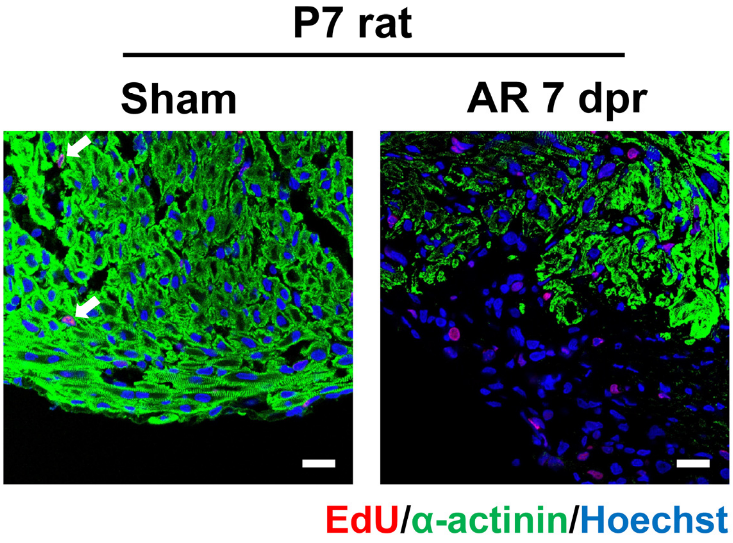

Supplementary Figure 12. Cardiac regenerative capacity in neonatal rats is lost from 7 days post birth. Representative images for co-immunofluorescent staining for α-actinin and EdU in sham- and apical resection (AR)-operated 7-day-old (P7) rat hearts at 7 days post resection (7 dpr) (n=6). There is no co-localization of α-actinin and EdU in the resected apex region of P7 AR rats. Scale bar=20 μm.

Supplementary Figure 13

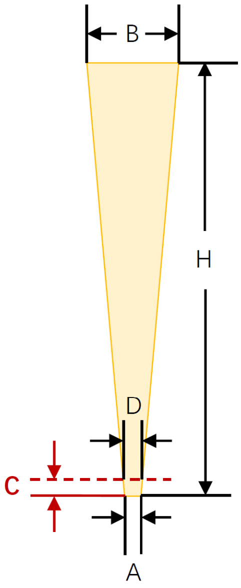

Length of **C** can be calculated by:

$$\frac{H}{B-A}=\frac{C}{D-A}$$

- D=0.48 mm to make a modified 10 μL pipette tip to perform AR in P1 mice
- D=0.63 mm to make a modified 200 μL pipette tip to perform AR in P1 rats

Supplementary Figure 13. Formular to calculate the distance cutting from the pipette tip to make a modified pipette tip with a given internal diameter. A and B represent the internal diameter of the tip and the base of pipette tip, respectively. H represents the height of the pipette tip. D represents the given internal diameter. C represents the distance cutting from the tip.

Supplementary Figure 14

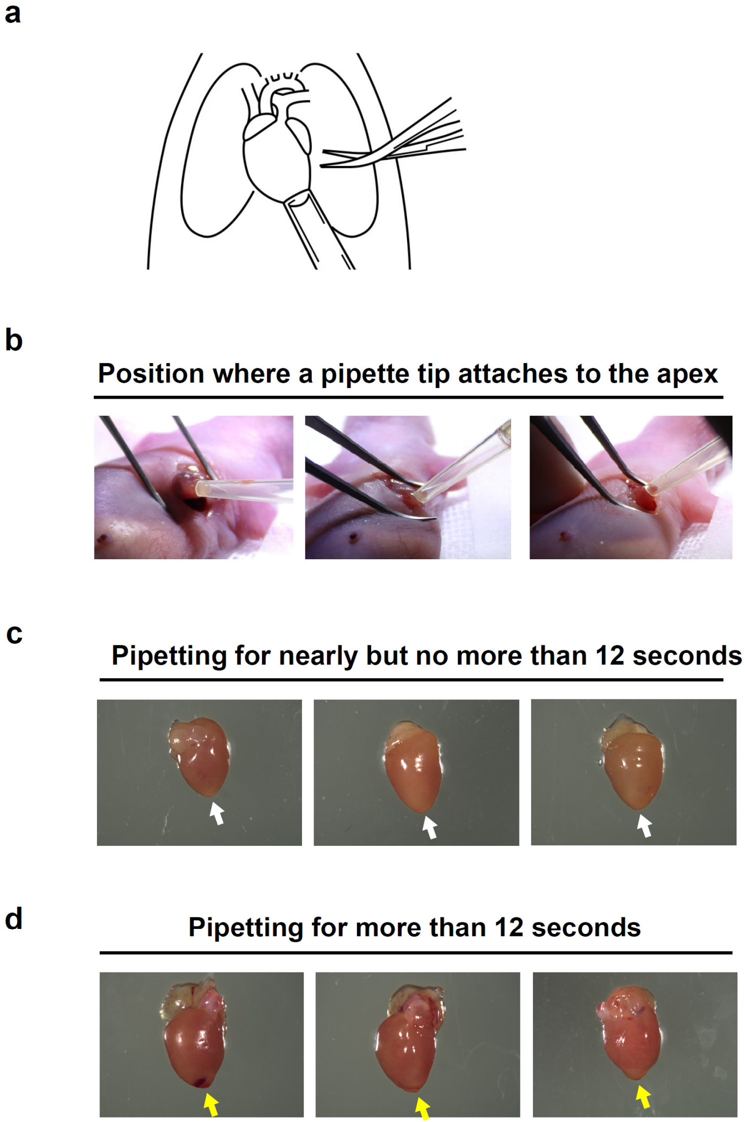

Supplementary Figure 14. A modified pipette tip attached to left ventricle apex of neonatal rats. (**a**) A schematic showing the precise position where a pipette tip attaches to left ventricle (LV) apex. (**b**) Photographs showing a modified 200 μL pipette tip attached to LV apex of 1-day-old neonatal rats. (**c** and **d**) Photographs taken for the whole hearts after pipette suction showing that a pipetting time nearly but no more than 12 seconds did not cause damage to the apex (**c**), while a pipetting time over 12 seconds risked to squeeze the apex and may cause damage to the apex (**d**).

Supplementary Figure 15

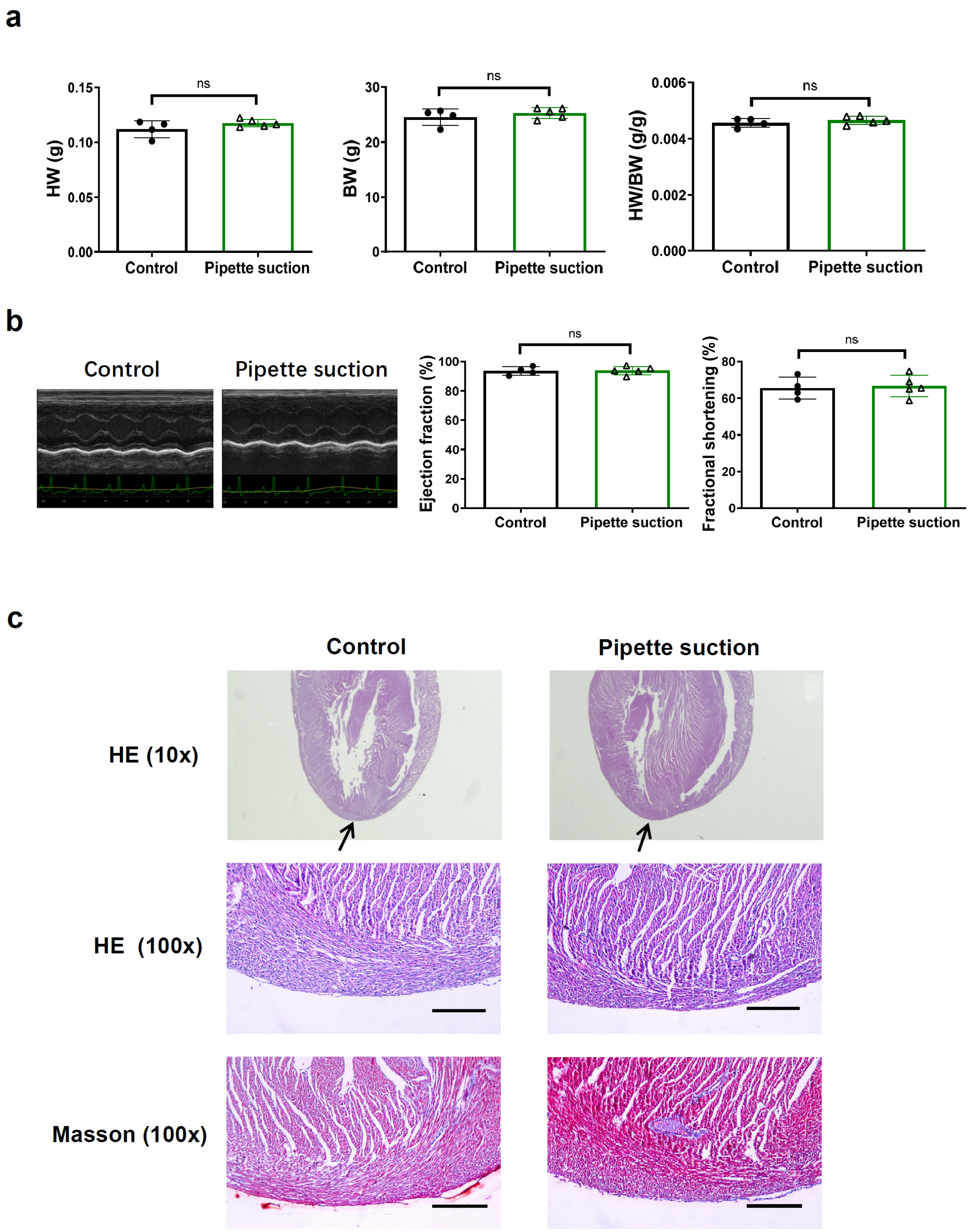

Supplementary Figure 15. Pipette suction for nearly 12 seconds does not cause damage to the heart. A pipette suction for nearly 12 seconds *versus* no pipette suction were performed in 1-day-old (P1) neonatal rats. (**a**) Heart weight (HW), body weight (BW), and heart weight/body weight ratio (HW/BW) at 7 days post operation (n=4-5). (**b**) Echocardiography for left ventricle ejection fraction and fractional shortening at 7

days post operation (n=4-5). (**c**) Representative images for hematoxylin-eosin (HE) and Masson's trichrome staining for hearts at 7 days post operation. Scale bar=200 μm. ns=not significant. All data are expressed as means±SD.

Supplementary Tables

Supplementary Table 1. Validation of 15% apical resection of whole heart weight in 1-day-old neonatal mice using modified AR method by 3 independent experiments.

Independent experiment	AR heart weight (g)	AR heart weight to intact heart weight (w/w, %)	Resected LV apex to intact heart weight (w/w, %)
1st experiment (n=5)	0.0069 ± 0.0004	84.7291	15.2709
2nd experiment (n=5)	0.0070 ± 0.0002	85.7843	14.2157
3rd experiment (n=5)	0.0061 ± 0.0002	85.3521	14.6479
Means ± SD	0.0066 ± 0.0005	85.2885 ± 0.4331	14.7115 ± 0.5305

AR=apical resection; LV=left ventricle. AR heart weight (g) indicates the mean value of the remaining heart weight after LV apex resection for each independent experiment.

Supplementary Table 2. Validation of 12% apical resection of whole heart weight in 1-day-old neonatal rats using modified AR method by 3 independent experiments.

Independent experiment	AR heart weight (g)	AR heart weight to intact heart weight (w/w, %)	Resected LV apex to intact heart weight (w/w, %)
1st experiment (n=7)	0.0352 ± 0.0040	87.0102	12.9898
2nd experiment (n=4)	0.0421 ± 0.0017	87.6955	12.3045
3rd experiment (n=3)	0.0401 ± 0.0029	88.4559	11.5441
Means ± SD	0.0382 ± 0.0045	87.7205 ± 0.5904	12.2795 ± 0.7231

AR=apical resection; LV=left ventricle. AR heart weight (g) indicates the mean value of the remaining heart weight after LV apex resection for each independent experiment.

Supplementary Table 3. Validation of 7% apical resection of whole heart weight in 1-day-old neonatal rats using modified AR method by 3 independent experiments.

Independent experiment	AR heart weight (g)	AR heart weight to intact heart weight	Resected LV apex to intact heart

		(w/w, %)	weight (w/w, %)
1st experiment (n=7)	0.0400 ± 0.0014	92.5877	7.4123
2nd experiment (n=3)	0.0451 ± 0.0020	93.6981	6.3019
3rd experiment (n=3)	0.0461 ± 0.0058	92.2563	7.7437
Means ± SD	0.0426 ± 0.0042	92.8474 ± 0.6166	7.1526 ± 0.7551

AR=apical resection; LV=left ventricle. AR heart weight (g) indicates the mean value of the remaining heart weight after LV apex resection for each independent experiment.

Supplementary Table 4. Validation of 5% apical resection of whole heart weight in 1-day-old neonatal rats using modified AR method by 3 independent experiments.

Independent experiment	AR heart weight (g)	AR heart weight to intact heart weight (w/w, %)	Resected LV apex to intact heart weight (w/w, %)
1st experiment (n=5)	0.0444 ± 0.0042	95.0385	4.9615
2nd experiment (n=5)	0.0413 ± 0.0027	94.5080	5.4920
3rd experiment (n=5)	0.0394 ± 0.0032	94.7115	5.2885
Means ± SD	0.0417 ± 0.0040	94.7527 ± 0.2185	5.2473 ± 0.2676

AR=apical resection; LV=left ventricle. AR heart weight (g) indicates the mean value of the remaining heart weight after LV apex resection for each independent experiment.

Supplementary Table 5. Primer sequences used for quantitative PCR.

Gene	Primer sequence
mmu-IL-1b-Forward	TGTGAAATGCCACCTTTTGA
mmu-IL-1b-Reverse	GGTCAAAGGTTTGGAAGCAG
mmu-IL-6-Forward	TGATGCACTTGCAGAAAACA
mmu-IL-6-Reverse	ACCAGAGGAAATTTTCAATAGGC
mmu-Ccl13-Forward	ACCATGACACTCTGCAACCA
mmu-Ccl13-Reverse	GTGGAATCTTCCGGCTGTAG
mmu-C5aR1-Forward	CAAGCTACAGCCCCAAAC
mmu-C5aR1-Reverse	GGGAGATGGTCACAGGAG
mmu-Mydgf-Forward	GGGTCGTGCATTCGTTCT

mmu-Mydgf-Reverse	TTGTCCCCAGGCTCATCT
mmu-Pitx2-Forward	AGGGAGGGAGGCAAGAAAAG
mmu-Pitx2-Reverse	CTTGAAAGAGCCAGGGAACG
mmu-18s-Forward	TCAAGAACGAAAGTCGGAGG
mmu-18s-Reverse	GGACATCTAAGGGCATCAC
rno-IL-1b-Forward	TGACAGGCAACCACTTACC
rno-IL-1b-Reverse	CCCATACACACGGACAACT
rno-IL-6-Forward	CACCAGGAACGAAAGTCAA
rno-IL-6-Reverse	CAACAACATCAGTCCCAAGA
rno-Ccl13-Forward	TGCCCTTGCTGTTCTTCT
rno-Ccl13-Reverse	AGGCTGCTGGTCTCAAAA
rno-C5aR1-Forward	TTCAAACCCATCTGGTGTC
rno-C5aR1-Reverse	AGGGGTCCTTATGTATCCG
rno-Mydgf-Forward	CAACGAGCAGTGGCAGA
rno-Mydgf-Reverse	GGCCTCCAGACGGTACA
rno-Pitx2-Forward	ACAATCTCCGATACGTCCA
rno-Pitx2-Reverse	TCCTCATTCTTTCCCTGCT
rno-18s-Forward	TCAAGAACGAAAGTCGGAGG
rno-18s-Reverse	GGACATCTAAGGGCATCAC
