# Supplementary material for: A modified apical resection model with high accuracy and reproducibility in neonatal mouse and rat hearts
Source: NPJ Regen Med. 2023 Feb 18;8:9. doi: 10.1038/s41536-023-00284-5 (PMC9938870; doi:10.1038/s41536-023-00284-5)
Supplement: Supplementary file 3 — Reporting Summary [file 41536_2023_284_MOESM3_ESM.pdf]

nature portfolio

Corresponding author(s): Yihua Bei & Junjie Xiao

Last updated by author(s): Jan 27, 2023

Reporting Summary

Nature Portfolio wishes to improve the reproducibility of the work that we publish. This form provides structure for consistency and transparency in reporting. For further information on Nature Portfolio policies, see our Editorial Policies and the Editorial Policy Checklist.

Statistics

For all statistical analyses, confirm that the following items are present in the figure legend, table legend, main text, or Methods section.

n/a	Confirmed	
☐	☒	The exact sample size (*n*) for each experimental group/condition, given as a discrete number and unit of measurement
☐	☒	A statement on whether measurements were taken from distinct samples or whether the same sample was measured repeatedly
☐	☒	The statistical test(s) used AND whether they are one- or two-sided<br>*Only common tests should be described solely by name; describe more complex techniques in the Methods section.*
☒	☐	A description of all covariates tested
☐	☒	A description of any assumptions or corrections, such as tests of normality and adjustment for multiple comparisons
☐	☒	A full description of the statistical parameters including central tendency (e.g. means) or other basic estimates (e.g. regression coefficient) AND variation (e.g. standard deviation) or associated estimates of uncertainty (e.g. confidence intervals)
☐	☒	For null hypothesis testing, the test statistic (e.g. *F*, *t*, *r*) with confidence intervals, effect sizes, degrees of freedom and *P* value noted<br>*Give P values as exact values whenever suitable.*
☒	☐	For Bayesian analysis, information on the choice of priors and Markov chain Monte Carlo settings
☒	☐	For hierarchical and complex designs, identification of the appropriate level for tests and full reporting of outcomes
☒	☐	Estimates of effect sizes (e.g. Cohen's *d*, Pearson's *r*), indicating how they were calculated

Our web collection on statistics for biologists contains articles on many of the points above.

Software and code

Policy information about availability of computer code

Data collection	Echocardiography was performed using the Vevo 2100 Imaging System (FUJIFILM Visual Sonics, Toronto, Ontario, Canada).<br>Histological stainings were observed and photographed under a biological microscope (Leica, DM3000).<br>Immunofluorescent staining images were viewed and taken under a confocal microscope (Zeiss, LSM710).<br>Quantitative polymerase chain reactions were performed on Roche LightCycler480 PCR System (Roche Diagnostics).<br>Western blot immunoblots were visualized using ChemiDoc MP Imaging System (Bio-Rad, 17001402) and Chemidoc Imaging System (Tanon, 5200S).
Data analysis	All data were analyzed using SPSS20.0 or GraphPad Prism8.

For manuscripts utilizing custom algorithms or software that are central to the research but not yet described in published literature, software must be made available to editors and reviewers. We strongly encourage code deposition in a community repository (e.g. GitHub). See the Nature Portfolio guidelines for submitting code & software for further information.

Data

Policy information about availability of data

All manuscripts must include a data availability statement. This statement should provide the following information, where applicable:

- Accession codes, unique identifiers, or web links for publicly available datasets
- A description of any restrictions on data availability
- For clinical datasets or third party data, please ensure that the statement adheres to our policy

All data generated or analysed during this study are included in this article and its supplementary information files.

Human research participants

Policy information about studies involving human research participants and Sex and Gender in Research.

Reporting on sex and gender	N/A
Population characteristics	N/A
Recruitment	N/A
Ethics oversight	N/A

Note that full information on the approval of the study protocol must also be provided in the manuscript.

Field-specific reporting

Please select the one below that is the best fit for your research. If you are not sure, read the appropriate sections before making your selection.

☒ Life sciences ☐ Behavioural & social sciences ☐ Ecological, evolutionary & environmental sciences

For a reference copy of the document with all sections, see nature.com/documents/nr-reporting-summary-flat.pdf

Life sciences study design

All studies must disclose on these points even when the disclosure is negative.

Sample size	No sample-size calculation was performed. Sample size were determined according to our previous animal experiments (PMID: 25863248, 35077859, 35674310). The numbers of sample sizes were described in each Figure Legend.
Data exclusions	No data were excluded from the analyses.
Replication	To evaluate the apical resection (AR) operation reproducibility, we trained 5 operators to perform both conventional and modified AR methods. Relative standard deviation (RSD) analysis was performed for evaluation of reproducibility of modified and conventional AR methods (Figure 3 and Table 1).<br>The weight of resected apex in proportion to whole heart weight was confirmed by 3 independent experiments (Table S1-S4).<br>All attempts at replication were successful.
Randomization	All animals were randomly assigned into experimental groups.
Blinding	The investigators for functional and structural measurements were blinded to group allocation during data collection and analysis.

Reporting for specific materials, systems and methods

We require information from authors about some types of materials, experimental systems and methods used in many studies. Here, indicate whether each material, system or method listed is relevant to your study. If you are not sure if a list item applies to your research, read the appropriate section before selecting a response.

Materials & experimental systems

n/a	Involved in the study
☐	☒ Antibodies
☒	☐ Eukaryotic cell lines
☒	☐ Palaeontology and archaeology
☐	☒ Animals and other organisms
☒	☐ Clinical data
☒	☐ Dual use research of concern

Methods

n/a	Involved in the study
☒	☐ ChIP-seq
☒	☐ Flow cytometry
☒	☐ MRI-based neuroimaging

Antibodies

Antibodies used

1. α-actinin (Sigma, A7811): Monoclonal (EA53) Anti-α-Actinin (Sarcomeric) antibody produced in mouse, lot: 0000152016.
2. pHH3 (Abclonal, AP0840): Polyclonal Anti-pHH3 antibody produced in Rabbit, lot: 3513473001.
3. Aurora B (Sigma, A5102): Polyclonal Anti-Aurora B antibody produced in rabbit, lot: 21181258.
4. p-YAP1-S127 (Abclonal, AP0489): Polyclonal Anti-p-YAP1-S127 antibody produced in Rabbit, lot: 5500000179.
5. YAP1 (Proteintech, 13584-1-AP): Polyclonal Anti-YAP1 antibody produced in Rabbit, lot: 00096348.
6. GAPDH (Bioworld, AP0063): Polyclonal Anti-GAPDH antibody produced in Rabbit, lot: 01201908.

Validation

1. α-actinin (Sigma, A7811): suitable for immunofluorescence applications in mouse and rat.
2. pHH3 (Abclonal, AP0840): suitable for immunofluorescence applications in mouse and rat.
3. Aurora B (Sigma, A5102): suitable for immunofluorescence applications in mouse and rat.
4. p-YAP1-S127 (Abclonal, AP0489): suitable for Western blot in mouse and rat.
5. YAP1 (Proteintech, 13584-1-AP): suitable for Western blot in mouse and rat.
6. GAPDH (Bioworld, AP0063): suitable for Western blot in mouse and rat.

Animals and other research organisms

Policy information about studies involving animals; ARRIVE guidelines recommended for reporting animal research, and Sex and Gender in Research

Laboratory animals

Eight-week-old C57BL/6J mice and Sprague-Dawley rats were purchased from Cavens Lab Animal (Changzhou, China) and maintained in the specific-pathogen-free (SPF) laboratory animal facility of Shanghai University (Shanghai, China). Their offspring was used to establish apical resection (AR) models in neonatal rodents.

Wild animals

No wild animals involved.

Reporting on sex

Since we performed apical resection (AR) surgery in neonatal rodents, both male and female neonates were used in our study.

Field-collected samples

No field-collected samples involved.

Ethics oversight

All animal experiments were conducted in concordance with the Guidelines for the Care and Use of Laboratory Animals for biomedical research published by the National Institutes of Health (No. 85-23, revised 1996) and approved by the Committee for the Ethics of Animal Experiments of Shanghai University.

Note that full information on the approval of the study protocol must also be provided in the manuscript.
